# Clinically Used Hormone Formulations Differentially Impact Memory, Anxiety-Like, and Depressive-Like Behaviors in a Rat Model of Transitional Menopause

**DOI:** 10.3389/fnbeh.2021.696838

**Published:** 2021-07-21

**Authors:** Stephanie V. Koebele, Ryoko Hiroi, Zachary M. T. Plumley, Ryan Melikian, Alesia V. Prakapenka, Shruti Patel, Catherine Carson, Destiney Kirby, Sarah E. Mennenga, Loretta P. Mayer, Cheryl A. Dyer, Heather A. Bimonte-Nelson

**Affiliations:** ^1^Department of Psychology, Arizona State University, Tempe, AZ, United States; ^2^Arizona Alzheimer’s Consortium, Phoenix, AZ, United States; ^3^FYXX Foundation, Flagstaff, AZ, United States

**Keywords:** VCD, menopause, estrogen, progesterone, levonorgestrel, memory, anxiety, depression

## Abstract

A variety of U.S. Food and Drug Administration-approved hormone therapy options are currently used to successfully alleviate unwanted symptoms associated with the changing endogenous hormonal milieu that occurs in midlife with menopause. Depending on the primary indication for treatment, different hormone therapy formulations are utilized, including estrogen-only, progestogen-only, or combined estrogen plus progestogen options. There is little known about how these formulations, or their unique pharmacodynamics, impact neurobiological processes. Seemingly disparate pre-clinical and clinical findings regarding the cognitive effects of hormone therapies, such as the negative effects associated with conjugated equine estrogens and medroxyprogesterone acetate vs. naturally circulating 17β-estradiol (E2) and progesterone, signal a critical need to further investigate the neuro-cognitive impact of hormone therapy formulations. Here, utilizing a rat model of transitional menopause, we administered either E2, progesterone, levonorgestrel, or combinations of E2 with progesterone or with levonorgestrel daily to follicle-depleted, middle-aged rats. A battery of assessments, including spatial memory, anxiety-like behaviors, and depressive-like behaviors, as well as endocrine status and ovarian follicle complement, were evaluated. Results indicate divergent outcomes for memory, anxiety, and depression, as well as unique physiological profiles, that were dependent upon the hormone regimen administered. Overall, the combination hormone treatments had the most consistently favorable profile for the domains evaluated in rats that had undergone experimentally induced transitional menopause and remained ovary-intact. The collective results underscore the importance of investigating variations in hormone therapy formulation as well as the menopause background upon which these formulations are delivered.

## Introduction

During the midlife transition to menopause, a number of symptoms that negatively impact quality of life and wellbeing may occur. Most commonly, these symptoms originate from natural changes in estrogen production by the ovaries as follicle reserve declines, leading to the onset of vasomotor symptoms (e.g., hot flashes, night sweats), dyspareunia, and urogenital indications (Hoffman et al., 2012; Al-Safi and Santoro, 2014; NAMS, 2014). Benign irregular or heavy bleeding patterns are also common during the transition to menopause (Voorhis et al., 2008; Corniţescu et al., 2011; Pinkerton, 2011). In addition, during the menopause transition many individuals report increased rates of depression and anxiety symptoms, as well as impaired cognition, particularly in the realm of working memory (Kritz-Silverstein et al., 2000; Mitchell and Woods, 2001; Weber and Mapstone, 2009; Maki et al., 2012; Weber et al., 2012, 2014; Worsley et al., 2014; Zilberman et al., 2015; Unkenstein et al., 2016; Rentz et al., 2017; Morgan et al., 2018; Im et al., 2019).

There are a variety of U.S. Food and Drug Administration (FDA)-approved hormone therapy options available that effectively alleviate undesirable symptoms associated with menopause-related changes in the endogenous hormonal milieu (Files et al., 2011; Hoffman et al., 2012; Pinkerton, 2012; Stuenkel et al., 2015; Pinkerton et al., 2017c). If the uterus is intact, a hormone therapy regimen must include a progestogen component (i.e., natural progesterone or one of the many synthetic forms of progesterone; the latter are collectively referred to as progestins) in combination with an estrogen component (e.g., natural 17β-estradiol (E2), synthetic ethinyl estradiol, conjugated equine estrogens). This progestogen component is necessary to mitigate the risk of uterine hyperplasia and cancer (Pinkerton et al., 2017c). If a patient’s primary indication for treatment is heavy, irregular, or abnormal uterine bleeding, medical professionals may prescribe a progestogen-only hormone therapy, such as an oral progestogen or an intrauterine device containing the progestin levonorgestrel, a synthetic form of progesterone (Sitruk-Ware, 2002; Marret et al., 2010; Corniţescu et al., 2011; Pinkerton, 2011; Goldstein and Lumsden, 2017). If a patient has undergone hysterectomy with or without ovary removal, they may take estrogen-only hormone therapy, as the removal of uterine tissue eliminates the need for the progestogen component (Haney and Wild, 2007; NAMS, 2014; Pinkerton et al., 2017c). Additionally, low-dose vaginal estrogen-only tablets, creams, and rings are increasing in popularity for the treatment of menopausal genitourinary syndrome even when the uterus is intact (Rahn et al., 2014; Pinkerton et al., 2017b; Biehl et al., 2018; Shifren, 2018). Thus, depending on an individual’s circumstance and primary indications for menopausal hormone therapy use, there are a range of possibilities for variations in hormone therapy preparations, including estrogen-only, progestogen-only, or combined estrogen plus progestogen hormone therapy options, which in turn may have variable effects on the brain and periphery.

Sex steroid hormones have been shown to impact learning and memory, although the ideal parameters for individual and combined hormone therapies have proven to be complex (for review, see: Barha and Galea, 2010; Gibbs, 2010; Luine, 2014; Frick, 2015; Koebele and Bimonte-Nelson, 2015, 2017; Korol and Pisani, 2015). Depriving the female system of ovarian-derived hormones leads to cognitive changes in both humans and animal models (e.g., Phillips and Sherwin, 1992; Singh et al., 1994; Bimonte and Denenberg, 1999; Nappi et al., 1999; Heikkinen et al., 2004; Wallace et al., 2006; Rocca et al., 2007; Gibbs and Johnson, 2008; Parker et al., 2009; Ryan et al., 2014). Importantly, ovarian hormone loss also results in an increased susceptibility to anxiety and depression (Parker et al., 2009; Bromberger and Kravitz, 2011; Bromberger et al., 2011; Maki et al., 2012; Weber et al., 2014; Parry, 2020; Soares, 2020; Stute et al., 2020). Under certain parameters or experimental conditions, estrogen supplementation following the surgical removal of the ovaries (ovariectomy; Ovx) reverses or attenuates detriments in cognition and affective behaviors in preclinical models (Bimonte and Denenberg, 1999; Holmes et al., 2002; Foster et al., 2003; Hiroi and Neumaier, 2006; Hiroi et al., 2006, 2016; Fernandez et al., 2008; Harburger et al., 2009; Rodgers et al., 2010; Gleason et al., 2015; Black et al., 2016, 2018; Koebele et al., 2020b). Much emphasis has been placed on exogenous E2 administration following Ovx, and reports show variable effects on cognition depending on the parameters. However, most individuals experience a natural, non-surgical transition to menopause and retain their ovaries. The ovatoxin 4-vinylcyclohexene diepoxide (VCD) induces accelerated follicular atresia, which serves as a rat model of transitional menopause, wherein ovarian tissue is maintained but becomes follicle-deplete (Mayer et al., 2002, 2004; Dyer et al., 2013; Koebele and Bimonte-Nelson, 2016). Using VCD, our laboratory recently demonstrated that compared to follicle-deplete rats that did not receive E2 treatment, tonic E2 had beneficial effects in the learning phase of a complex spatial working memory task. However, some working memory impairments were evident in the E2-treated rats after the rules of the task had been acquired (Koebele et al., 2020a), demonstrating the complex role of estrogens in learning and memory.

Although E2 is a common component in many FDA-approved combined hormone therapy formulations, the progestogen component varies. Progestins are used frequently as an alternative to natural progesterone due to significantly higher oral bioavailability (Sitruk-Ware et al., 1987; Schindler et al., 2003; Kuhl, 2005). All progestins exert progestogenic activity at progesterone receptors, resulting in protective mechanisms for the uterus, which is often their primary clinical application. However, depending on its molecular derivative, a given progestin can also have estrogenic, anti-estrogenic, androgenic, anti-androgenic, and/or glucocorticoid activity to varying extents (Schindler et al., 2003). These unique pharmacological profiles lead to distinct patterns of activity and actions by progestins, including variable cognitive effects (Sitruk-Ware, 2002; Schindler et al., 2003; Braden et al., 2017). Several progestins have been shown by our and other laboratories to negatively affect cognition (Rapp et al., 2003; Shumaker et al., 2003; Rosario et al., 2006; Braden et al., 2010, 2011; Lowry et al., 2010). However, levonorgestrel, a common progestin in hormone therapy formulations and a hormone-containing intrauterine device, has been reported to have neutral, or even beneficial, effects on cognition in the surgical menopause (i.e., Ovx) rat model when administered independently (Braden et al., 2017; Prakapenka et al., 2018). Levonorgestrel may exhibit these unique effects due to its distinct pharmacodynamic properties; in contrast to natural progesterone or other progestins, levonorgestrel does not elicit glucocorticoid or anti-mineralocorticoid receptor activity, but does have some androgenic activity (Schindler et al., 2003). For example, in middle-aged Ovx rats, we have demonstrated that levonorgestrel alone produced cognitive benefits; however, when levonorgestrel was co-administered with E2, it failed to augment, and in fact attenuated, E2’s favorable effects on cognition, producing impairments relative to either hormone alone (Prakapenka et al., 2018). These results highlight the importance of performing translational research in which clinical practices are accurately modeled. Whether a combined E2 + progestogen regimen exerts similar effects in a model of transitional menopause remains to be determined. This is a question of high importance, given that minor alterations in molecular structure can lead to different physiological effects of progestogens (Sitruk-Ware, 2002), and that progestogens are most often given in combination with E2 when an individual undergoing menopause has an intact uterus and ovaries (Pinkerton et al., 2017c). It is critical to methodically compare how daily administration of natural progesterone and the progestin levonorgestrel influence learning and memory independently as well as in combination with E2, and whether progestogen type matters for outcomes with transitional menopause.

To address this question, we administered VCD to permit the retention of follicle-depleted ovarian tissue and to produce a circulating hormone profile more similar to that associated with transitional menopause than would be achievable with Ovx (Koebele and Bimonte-Nelson, 2016). In the current experiment, VCD treatment began at 8 months of age, as we have done in previous publications (Koebele et al., 2020a). Three months later, when rats were middle-aged and considered to be in the early post-menopausal stage after substantial follicular depletion ensued (Lohff et al., 2005; Acosta et al., 2009; Koebele et al., 2020a), daily exogenous hormone treatment began and rats were tested on a behavioral battery assessing spatial memory, anxiety-like, and depressive-like behaviors. Thus, the goals of the current experiment were manifold, as we aimed to systematically evaluate the independent and combined effects of daily E2, progesterone, and levonorgestrel on cognitive, anxiety-like, and depressive-like measures in transitionally menopausal, follicle-deplete, middle-aged rats.

## Materials and Methods

**FIGURE 1:**
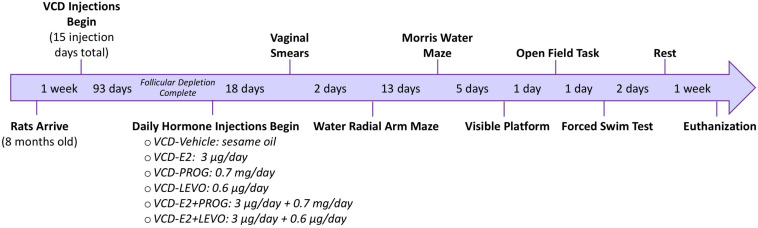
Experimental Timeline. Following accelerated follicular depletion, rats received daily hormone treatments and were evaluated on a series of behavior tasks assessing working memory, reference memory, anxiety-like behavior, and depressive-like behavior.

See Figure 1 for a detailed experimental timeline.

### Subjects

Sixty sexually inexperienced female Fischer-344-CDF rats from the National Institute on Aging colony at Charles River Laboratories (Raleigh, NC) were used in this experiment. Rats were approximately 8 months of age when they arrived at the Arizona State University vivarium facility. Rats were pair-housed upon arrival and had unrestricted access to food and water for the duration of the experiment. Rats were maintained on a 12-h light/dark cycle (lights on at 7 am) and had a 1 week period of acclimation in the vivarium prior to the commencement of experimental procedures. The Institutional Animal Care and Use Committee at Arizona State University approved all procedures, which adhered to National Institutes of Health standards.

### VCD Injections

All rats were administered VCD (FYXX Foundation, Flagstaff, AZ) intraperitoneally at a dose of 160 mg/kg/day in 50% dimethyl sulfoxide (DMSO)/50% sterile saline vehicle solution (Sigma-Aldrich, St. Louis, MO, United States) for a total of 15 injection days, based on established protocols (Mayer et al., 2002, 2004; Lohff et al., 2005, 2006; Acosta et al., 2009, 2010; Van Kempen et al., 2011; Frye et al., 2012; Zhang et al., 2016; Koebele et al., 2017, 2020a; Kirshner and Gibbs, 2018; Carolino et al., 2019). Baseline body weight (g) was recorded for all subjects prior to starting injections. VCD injection volume was calculated based on individual daily body weight. If a rat’s body weight decreased by 10% or more from its baseline, VCD administration was discontinued until weight was recovered. VCD was administered on Mondays, Tuesdays, Thursdays, and Fridays. Injections were not administered on Wednesdays, Saturdays, or Sundays for weight recovery (Koebele et al., 2017, 2020a). As such, to accommodate injection-related weight loss and recovery, the 15 VCD injections were completed over approximately 9 weeks. Two rats died during VCD injections: one from peritonitis and one from an undetermined cause, likely unrelated to injections.

### Hormone Treatment Administration

A 93 day waiting period from the first VCD injection was employed to ensure substantial ovarian follicular depletion (Lohff et al., 2005; Acosta et al., 2009; Koebele et al., 2020a) prior to initiating daily hormone administration, modeling the early post-menopausal time point. Rats were then randomly assigned to one of the following treatment conditions: Vehicle (sesame oil; Sigma Aldrich S3547; *n* = 10), 17β-estradiol (E2; 3 μg/day; Sigma Aldrich, E8875; *n* = 10), Progesterone (PROG; 0.7 mg/day; Sigma Aldrich, P0130; *n* = 9), Levonorgestrel (LEVO; 0.6 μg/day; Sigma Aldrich, N2260; *n* = 9), E2 + PROG (3 μg E2 + 0.7 mg PROG/day; *n* = 10), or E2 + LEVO (3 μg E2 + 0.6 μg LEVO/day; *n* = 10) (summarized in Figure 1). All hormone treatments were dissolved in sesame oil, delivered via a 0.10 mL daily subcutaneous injection for 21 days prior to beginning behavioral testing. Hormone or Vehicle injections continued for the duration of the experiment until euthanasia.

### Body Weights

Body weights (g) were recorded for all rats at the onset of VCD injections and periodically collected throughout the experiment until euthanasia. Body weight served as a peripheral indicator of general animal health and was used to assess whether hormone treatments altered body weight in an ovary-intact, follicle-depleted background.

### Vaginal Cytology

Vaginal smears were assessed immediately prior to behavioral testing initiation for two consecutive days, as previously published (Koebele et al., 2020a). The experimenter obtained each swab sample by gently inserting a small cotton-tipped applicator soaked in sterile saline into the vaginal opening. A light microscope (Fisher Scientific Micromaster; CAT #12-561-4B) was used to view the cells at 10× magnification. The experimenter classified samples as proestrus-, estrus-, metestrus-, or diestrus- like as our laboratory and others have previously published (Goldman et al., 2007; Koebele and Bimonte-Nelson, 2016; Koebele et al., 2019).

### Behavioral Testing

After 3 weeks of daily hormone administration, 114 days after the first VCD injection, all rats (approximately 11–12 months old) were tested on a series of behavioral tasks assessing spatial working and reference memory, anxiety-like behavior, and depressive-like behavior. These assays included the water radial arm maze (WRAM) to evaluate spatial working and reference memory, the Morris water maze (MM) to assess spatial reference memory, the visible platform (VP) task to confirm motor and visual competency for swim-based tasks, the open field task (OFT) to assess locomotor activity and anxiety-like behavior, and the forced swim task (FST) to evaluate depressive-like behavior. Procedures for each task are described in detail below.

#### Water Radial Arm Maze

The WRAM evaluated spatial working and reference memory in a water escape paradigm (Bimonte-Nelson et al., 2015). The apparatus had eight arms (38.1 cm × 12.7 cm each) and a circular center, and was filled with water maintained at 18–20°C throughout testing. To assist with spatial navigation, prominent visual cues were placed on the walls around the maze in addition to the tables and heat lamps situated in each room. A pre-selected combination of platform locations was assigned to each rat, wherein hidden escape platforms were submerged 2–3 cm beneath the water’s surface in four of the eight maze arms (locations counterbalanced across treatment groups); the other four arms (including the start arm) never contained platforms. Assigned platform locations remained the same across all testing days for a given rat. Black non-toxic powdered paint was added to the water to further obscure submerged platforms. Testing consisted of four trials per day across 13 consecutive days. Day 1 was considered training, days 2–12 were normal testing sessions, and day 13 included a delayed memory retention evaluation. During each daily testing session, the experimenter gently placed the rat in the non-platformed start arm. If the rat did not escape the WRAM via a hidden platform within the allotted 3-min trial time, the experimenter guided the rat to the nearest platform using a lead stick. Upon locating a platform, the rat was allocated 15 s of total platform time before being returned to its heated testing cage to reinforce platform location learning. During a 30 s inter-trial-interval (ITI), the experimenter removed the just-found platform from the maze, swept the water for debris with a net, and stirred the water to diffuse potential olfactory cues. In this way, working memory load progressively became taxed across trials within a daily testing session, as the number of locations to be recalled increased with each trial. On Day 13 of testing, a 6-h delay was implemented between trials two and three to assess delayed working memory retention. During the delay interval, rats were kept in their individual testing cages and given access to water.

Learning and memory performance on the WRAM was quantified by calculating the number of entries into non-platformed arms prior to locating a platform on each trial within a day, which were considered errors. The experimenter logged each arm entry error manually on a testing sheet during the trials. An entry was operationally defined as the tip of the rat’s snout crossing a marker 11 cm into the arm (visible on the outside of the maze, but not visible to the rat). Errors were counted and divided into subtypes. Working memory correct (WMC) errors were entries into an arm that previously contained a hidden platform within a daily testing session. Of note, WMC errors can only occur on trials 2–4, as all platforms are present in the maze during the first trial; as such, statistical analyses for WMC errors across trials are inclusive of trials 2, 3, and 4. Reference memory (RM) errors were the first entries into an arm within a daily testing session that never contained a platform; as such, a total of four RM errors could be made within a daily testing session. Working memory incorrect (WMI) errors were subsequent entries, within a daily testing session, into an arm that never contained a platform (Bimonte-Nelson et al., 2015). RM and WMI errors can be made on any trial; thus, analyses for WMI and RM errors across trials are inclusive of trials 1–4.

#### Morris Water Maze

Following the WRAM delayed memory retention day, rats were evaluated on the MM, a water-escape task which assesses spatial reference memory (Morris et al., 1982; Morris, 2015). The MM was a circular tub (188 cm in diameter) filled with 18–20°C water made opaque with non-toxic black paint. One platform (11 cm diameter) was placed 2–3 cm below the surface of the water in the northeast quadrant of the tub, where it remained across all days and trials. The rats underwent four trials per day for five consecutive days. During each daily session, rats were dropped off from one of four directions (north, south, east, or west) at the start of each trial. The pattern of the four drop-off locations changed across days but was identical within a day for all rats. Path length (cm) from drop-off to the platform was recorded by a video camera and Ethovision tracking software (Noldus Instruments; Wageningen, Netherlands). Maximum trial time was capped at 1 min. If the rat did not navigate to the platform in the allotted trial time, the experimenter gently guided the rat to the platform using a lead stick. Once the rat located the hidden platform, it was required to stay there for 15 s of platform time before being returned to its heated testing cage for a ∼10-min ITI, during which the other subjects were tested on that trial. On the final testing day of MM, after the fourth trial, rats completed a probe trial wherein the submerged platform was completely removed from the maze. Rats swam freely in the maze for the 1-min probe trial. The proportion of total swim distance covered within the previously platformed quadrant vs. the opposite quadrant was calculated to assess spatial localization to the previous platform location.

#### Visible Platform

On the day following MM, generalized visual acuity and motor competency necessary for completing swim-based escape tasks were assessed using the VP control task (Morris, 1984; Mennenga et al., 2015a). The VP was a rectangular tub (100 cm × 60 cm) filled with clear water (18–20°C). On the north wall of the tub, a black platform (10 cm diameter) protruded approximately 4 cm above the water’s surface and was easily visible to the rats. Opaque curtains surrounded the VP apparatus to obscure spatial and geometric cues within the testing room. Rats underwent six trials in 1 day. Each rat was dropped off from a fixed location in the center of the south wall of the tub. The platform position varied across trials semi-randomly in three possible locations along the north wall: left, center, and right. Each trial was capped at 90 s to reach the visible platform. The experimenter used a stopwatch to obtain latency to the platform and recorded it manually on a testing sheet after each trial. After navigating to the visible platform, the rat was required to stay on the platform for 15 s before the experimenter returned the rat to its heated testing cage outside of the opaque curtains. There was an ITI of approximately 10 min for each rat while the other subjects were tested on that trial.

#### Open Field Task

The day after VP, rats underwent one evaluation day in the OFT, which measured locomotor activity and anxiety-like behavior. Twenty-four hours before testing, the 100 cm × 100 cm black Plexiglas arena was thoroughly cleaned with Odormute, an enzyme cleaner, to remove potential odors from the apparatus. OFT procedures were carried out in a dark room, a protocol which has previously been found to be sensitive to changes in hormone profiles in female rats (Hiroi and Neumaier, 2006; Hiroi et al., 2016). At the beginning of the testing day, rats were transferred from their home cages to single testing cages and allowed to acclimate in the anteroom of the testing area for at least 30 min. Each subject was brought into the room separately. The experimenter placed the rat into the arena along the center of the north wall and quietly exited the room. Each rat had 10 min to freely explore the arena. Trials were recorded using Samsung infrared night vision cameras connected to an iPad via the SmartCam application. Following each trial, the experimenter reentered the room, removed the rat from the arena, discarded any feces or urine in the arena, and wiped down the entire arena with tap water to distribute odor cues. The box was dried with paper towel prior to the beginning of the next subject’s trial. Using an overlay of 25 evenly sized and shaped squares (20 cm × 20 cm), an experimenter blind to treatment conditions manually scored the recorded trials for time spent (s) in the corners, center, and small center of the arena, as well as line crossings into the corners, center, small center, and total line crossings.

#### Forced Swim Task

The day following the OFT, rats were exposed to 2 days of the FST to evaluate depressive-like behaviors (Huynh et al., 2011; Hiroi et al., 2016). Four clear Plexiglas cylinders (45 cm high and 20 cm in diameter) were filled up to 30 cm in height with fresh water (25°C) and separated by black Plexiglas divider screens. On day one of the FST, rats were acclimated to the testing room for at least 30 min. Each rat was placed in a cylinder for 10 min before being removed, toweled dry, and placed back into a heated testing cage. Twenty-four hours later, rats were given a 5-min trial under the same conditions. Video recordings of the 5-min trial on day two were captured using a GoPro camera connected to an iPad. After the trial was completed, rats were removed from the cylinder and towel dried prior to being placed under an escapable heat lamp. Number of fecal boli were recorded after the trial. The water was drained from the clear cylinder and refilled with fresh water between each subject’s trial. Recordings were scored by an independent experimenter blind to treatment conditions for latency to first immobility (s), time immobile (s), time climbing (s), time swimming (s), and number of dives. Immobility was quantified as minor movements necessary to keep the rat’s head above water. Climbing was scored as rapid forearm movement to break the surface of the water or upward vertical movement to climb against the cylinder wall. Diving was defined as a rapid downward movement into the cylinder. Any other motion made by the rats during the 5-min trial was identified as swimming behavior.

### Euthanasia

Rats were given 1 week of rest following the FST prior to euthanasia. At approximately 13 months old, all subjects were deeply anesthetized using inhaled isoflurane prior to cardiocentesis and decapitation. Blood was collected from the left ventricle of the heart using a 20 g needle and allowed to clot at 4°C (Vacutainer 367986; Becton Dickinson and Company, Franklin Lakes, NJ, United States) for a minimum of 30 min. Blood vials were maintained on ice and centrifuged at 2000 rpm at 4°C for 20 min at the end of the day. Serum was aliquoted and stored at −20°C until analysis. Ovaries were separated from the uterine horns, trimmed of excess fat, and fixed in 10% buffered formalin for 48 h prior to being transferred to 70% ethanol until analysis. Uteri were dissected from the body cavity, trimmed of excess fat, and wet weight (g) was obtained.

### Serum Hormone Measurements

All serum hormone assay processing was completed at the Core Endocrine Laboratory at Pennsylvania State University. E2 levels were detected using a double antibody liquid-phase radioimmunoassay (Beckman Coulter, Brea, CA, United States) as previously described (Acosta et al., 2010; Camp et al., 2012; Mennenga et al., 2015b,c; Koebele et al., 2017, 2019). This RIA used estradiol-specific antibodies with a ^125^I-labeled estradiol as the tracer. Inter-assay coefficients of variation for the assay averaged 10% at a mean value of 28 pg/ml. E2 assay functional sensitivity was 5 pg/ml. Androstenedione levels were evaluated via ELISA (ALPCO, Salem, NH, United States) based on the typical competitive binding scenario between unlabeled antigen (present in standards, controls, and unknowns) and the enzyme-labeled antigen (conjugate) for a limited number of antibody binding sites on the microwell plate. Inter-assay coefficients of variation for the androstenedione assay averaged 9% at a mean value of 0.5 ng/ml. Functional sensitivity of the androstenedione assay was 0.1 ng/ml. Progesterone levels were also evaluated using ELISA (ALPCO, Salem, NH, United States). Progesterone ELISA inter-assay coefficients of variation averaged 13% at a mean value of 2.6 ng/ml. Functional sensitivity of the progesterone assay was 0.3 ng/ml.

### Ovarian Follicle Counts

Following post-fixation at euthanasia, one ovary from each rat was randomly selected for processing and quantification. All ovarian follicle histology and quantification was carried out by FYXX Foundation (Flagstaff, AZ, United States). The oviduct was separated from the ovary prior to processing by a Leica TP1020 tissue processor. The ovary was paraffin embedded and serial sectioned at 5 μm on a semi-automatic rotary microtome. Every 10th section was placed on slides, which were stained with Gills 2 hematoxylin and counterstained with eosin Y-phloxine B, then manually cover-slipped. Tissue was scanned for analysis using a 3D HisTech DESK Scanner. Every 20th section was analyzed for viable primordial, primary, secondary and antral follicles. Viable follicles were those with no apparent signs of atresia. Atretic follicles were not counted. Criteria from Haas et al. (2007) was used to classify follicle type. Briefly, a resting-state primordial cell was classified by a single layer of squamous granulosa cells around an oocyte. Primary follicles included a single layer of cuboidal granulosa cells. Secondary follicles were identified by several layers of granulosa cells surrounding the oocyte. Antral follicles had two or more layers of granulosa cells in addition to a fluid-filled antral space within the follicle (Haas et al., 2007). The estimated total number of primordial follicles was obtained using the following formula: N_t_ = (N_0_ × S_t_ × t_*s*_)/(S_0_ × d_0_), where N_t_ = total follicle estimate, N_0_ = number of follicles observed in the ovary, S_t_ = total number of sections in the ovary, t_*s*_ = thickness of the section (μm), S_0_ = total number of sections observed, and d_0_ = mean diameter of the nucleus (Gougeon and Chainy, 1987). Counts for primary, secondary, and antral follicles were summed. Corpora lutea were counted through progression of appearance across the entire sample.

### Statistical Analyses

Statview statistical software was used to complete data analyses. All analyses were two-tailed (α = 0.05) and presented as means ± S.E.M. A series of two-group planned comparison repeated measures ANOVAs were completed using Treatment as the independent variable. We aimed to answer three key questions with our experimental data. We asked: *(1) What role does daily E2-only treatment have with transitional menopause?* For this question, the VCD-E2 group was compared to the VCD-Vehicle group. *(2) Does daily treatment with an individual progestogen impact cognition with transitional menopause, and is type of progestogen a factor for outcomes?* To address this question, we compared the VCD-Vehicle group to the VCD-PROG group and to the VCD-LEVO group, as well as the VCD-PROG group to the VCD-LEVO group. *(3) What role does combination hormone therapy play for cognition with transitional menopause?* The VCD-E2 group was compared to each combination group (VCD-E2 + PROG and VCD-E2 + LEVO) to assess the impact of adding a progestogen component to E2 therapy in a reproductive tract intact, but follicle-deplete, system. The VCD-PROG and VCD-LEVO groups were compared to their corresponding combination hormone treatment groups (VCD-E2 + PROG or VCD-E2 + LEVO, respectively) to understand how E2 alters progestogen-only effects in a reproductive tract intact, but follicle-deplete, system. Combination groups were also compared to the VCD-Vehicle group, and to each other to evaluate whether different progestogen components of combined hormone therapy matter for cognitive outcomes. Statistically significant two-group comparisons are reported herein, while select non-significant comparisons key to the highlighted questions are provided for context.

Water radial arm maze data were divided into three phase blocks, as previously published (Mennenga et al., 2015c; Braden et al., 2017; Prakapenka et al., 2018; Koebele et al., 2019). Day 1 was considered training and was excluded from the analysis. Days 2–5 were the Early Acquisition Phase, Days 6–9 the Late Acquisition Phase, and Days 10–12 the Asymptotic Phase. Each phase block was analyzed separately, and each error type was analyzed separately for each phase block, with WMC, WMI, and RM errors as the dependent measures. The three trials for WMC, or four trials for WMI and RM, were nested within days within each phase block (Early Acquisition Phase Block 1: 4 days, Late Acquisition Phase Block 2: 4 days, Asymptotic Phase Block 3: 3 days) as the repeated measures. Thus, these analyses consisted of two-group ANOVAs with Treatment as the independent variable, and two repeated measures variables of trials within days (Trials), and days within block (Days). Separate *a priori* two-group analyses were run for Trial 3 + Trial 4, the high working memory load trials, for WMC and WMI errors on each block based on prior age- and hormone-mediated effects found in our laboratory (Bimonte and Denenberg, 1999; Bimonte et al., 2003; Bimonte-Nelson et al., 2003, 2004; Acosta et al., 2010; Mennenga et al., 2015b,c; Koebele et al., 2017, 2019, 2020b; Prakapenka et al., 2018). Delayed memory retention data were analyzed independently for each treatment group by comparing WMC errors on Trial 3 on the last day of regular testing to Trial 3 on Day 13, the first post-delay trial on the Delay Day.

Morris water maze analyses were completed using the same two-group comparison structure. Swim Distance to the Platform (cm) was the dependent measure, and the four trials per day were nested within the 5 days of the task as the repeated measures. Performance was assessed across all 5 days of the task as well as across the four regular (non-probe trial) trials on Day 5 alone. Probe trial data were analyzed for each treatment group using Proportion Total Swim Distance in the NE (target) vs. SW (opposite) quadrants.

Visible platform analyses were completed for individual treatment groups. Analyses comparing performance on Trial 1 to Trial 6 were compared within each group. Latency to Platform (s) was the dependent measure, and the first and last trials were repeated measures.

Open field task analyses were completed for each two-group comparison. ANOVA was used to analyze total time (s) spent in the corners, center, and small center of the arena, as well as total number of entries made into the corner, center, and small centers of the arena to assess anxiety-like behavior. The total number of line crossings were assessed to evaluate locomotor activity during the task. The number of fecal boli produced during the 10 min trial was quantified.

Forced swim task analyses were completed for each two-group comparison. ANOVA was used to analyze latency to first immobility (s), total immobility duration (s), total swimming duration (s), total climbing duration (s), and number of dives as measures of depressive-like behaviors, as well as the number of fecal boli produced during the trial.

Body weights, uterine weights, serum hormone levels, and ovarian follicle counts were analyzed using ANOVA. For each two-group comparison, Treatment was the independent variable and body weight (g), uterine weight (g), hormone levels (pg/mL or ng/mL), or follicle counts were the dependent measures. An additional set of analyses for ovarian follicle counts were carried out *post hoc* to include a comparison group of ovary-intact, non-VCD treated rats from an independent data set in our laboratory quantified by FYXX Foundation (*n* = 10). This ovary-intact group received the respective Vehicle injection (50%DMSO/50%Saline) for VCD injections to provide additional context for the VCD-induced follicular depletion in the current study. Unless otherwise noted, the number of subjects per treatment group in the reported analyses was as follows: VCD-Vehicle *n* = 10, VCD-E2 *n* = 10, VCD-PROG *n* = 9, VCD-LEVO *n* = 9, E2 + PROG *n* = 10, and E2 + LEVO *n* = 10.

## Results

### Water Radial Arm Maze

**FIGURE 2:**
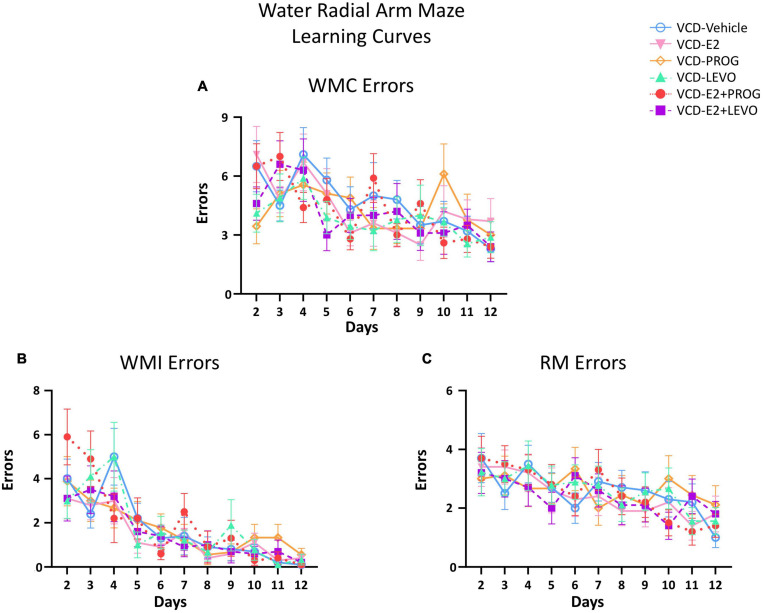
Water Radial Arm Maze Error Subtype Learning Curves. **(A)** Working memory correct errors across days **(B)** Working memory incorrect errors across days **(C)** Reference memory errors across days. For all error types, Day 1 was considered Training and was excluded from data analysis. The Early Acquisition Phase was defined as Days 2–5, the Late Acquisition Phase was defined as Days 6–9, and the Asymptotic Phase was defined as Days 10–12. Performance for each error subtype was analyzed separately. The n/group for all WRAM two-group analyses were: VCD-Vehicle *n* = 10, VCD-E2 *n* = 10, VCD-PROG *n* = 9, VCD-LEVO *n* = 9, VCD-E2 + PROG *n* = 10, and VCD-E2 + LEVO *n* = 10.

Figure 2 illustrates the learning curves for WMC (Figure 2A), WMI (Figure 2B), and RM (Figure 2C) errors across Days 2–12 of the WRAM.

#### Early Acquisition Phase (Days 2–5)

##### What role does daily E2-only treatment have in spatial learning and memory with transitional menopause?

The VCD E2 vs. VCD-Vehicle groups did not differ for WMC, WMI, or RM errors during the Early Acquisition Phase, suggesting that daily E2 treatment at the given dose did not affect early task learning in a model of transitional menopause compared to follicle-depleted rats that did not receive hormone treatment.

##### Does daily treatment with an individual progestogen impact cognition with transitional menopause, and does type of progestogen impact outcomes?

There were no differences between the VCD-Vehicle group and the VCD-PROG group or the VCD-LEVO group, nor between the VCD-PROG vs. VCD-LEVO groups for WMC, WMI, or RM during the Early Acquisition Phase. This suggests that with transitional menopause, daily progestogen treatment does not influence early task learning as compared to no hormone treatment, nor does type of progestogen differentially impact outcomes during learning.

##### What role does daily combination hormone therapy play for spatial learning and memory with transitional menopause?

**FIGURE 3:**
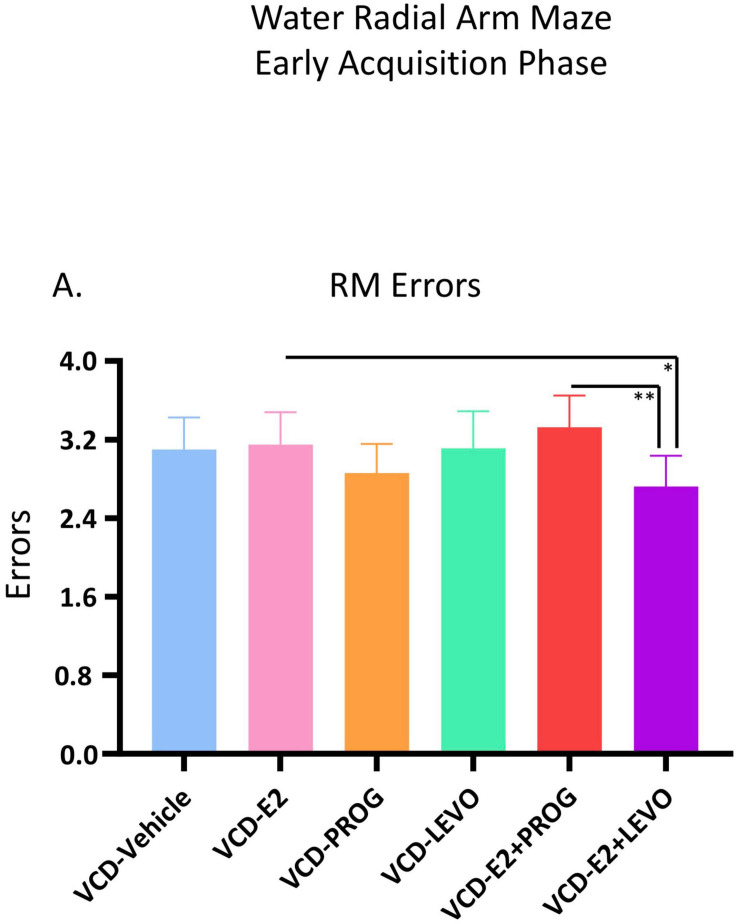
Early Acquisition Phase RM Errors Across All Trials (Two-Group Comparisons). The VCD-E2 + LEVO group showed enhanced reference memory performance compared to the VCD-E2 group (*p* < 0.05) and compared to the VCD-E2 + PROG group (*p* < 0.01) during the Early Acquisition Phase. Significance: ^∗^ = *p* < 0.05, ^∗∗^ = *p* < 0.01.

For RM errors, there was a main effect of Treatment for the VCD-E2 vs. VCD-E2 + LEVO comparison [*F*_(__1_,_18__)_ = 4.54, *p* < 0.05], where follicle-deplete rats treated with a combination of E2 and levonorgestrel made fewer RM errors compared to those treated with E2-only (Figure 3). For the VCD-E2 + PROG group vs. VCD-E2 + LEVO group, there was also a main effect [*F*_(__1_,_18__)_ = 9.78, *p* < 0.01], where follicle-deplete rats treated with a combination of E2 plus levonorgestrel made fewer RM errors than those treated with a combination of E2 plus progesterone during the Early Acquisition Phase. Thus, a daily regimen of E2 plus levonorgestrel combined with transitional menopause may confer benefits to spatial reference memory performance during learning (Figure 3).

#### Late Acquisition Phase (Days 6–9)

There were no significant Treatment differences in WMC, WMI, or RM errors for any two-group comparison during the Late Acquisition Phase.

#### Asymptotic Phase (Days 10–12)

##### What role does daily E2-only treatment have in spatial learning and memory with transitional menopause?

There were no significant differences in WMC, WMI, or RM errors for the VCD-E2 vs. VCD-Vehicle group comparison during the Asymptotic Phase of testing (Figures 5, 6), suggesting that daily E2 treatment at the given dose did not significantly affect memory maintenance with transitional menopause compared to counterparts that did not receive hormone treatment.

##### Does daily treatment with an individual progestogen impact cognition with transitional menopause, and is type of progestogen a factor for spatial learning and memory?

**FIGURE 4:**
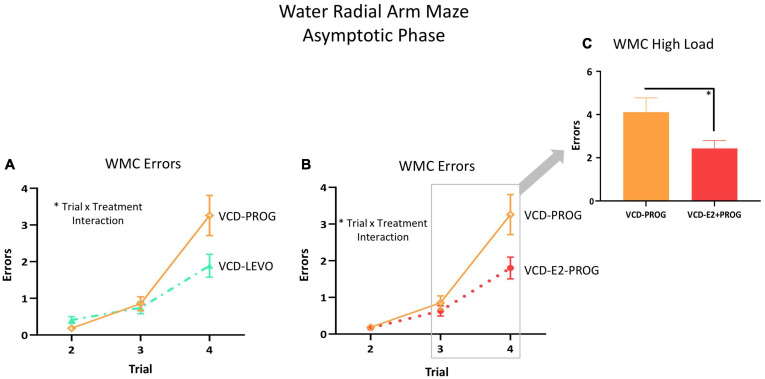
WMC Errors During the Asymptotic Phase. **(A)** While there was no main effect of Treatment collapsed across trials for any two-group comparison, there were Trial × Treatment interactions between the VCD-LEVO and VCD-PROG groups (*p* < 0.05) and **(B)** VCD-PROG and VCD-E2 + PROG groups (*p* < 0.05). **(C)** When the High Load trials (Trials 3 plus 4) were assessed, VCD-PROG rats made more WMC errors than VCD-E2 + PROG rats (*p* < 0.05). Significance: ^∗^ = *p* < 0.05.

During the Asymptotic Phase, there were no main effects of Treatment for WMC errors. There was a Trial × Treatment interaction present for WMC errors where follicle-deplete rats treated with progesterone performed worse than those treated with levonorgestrel (VCD-PROG vs. VCD-LEVO: *F*_(__2_,_32__)_ = 3.76, *p* < 0.05; Figure 4A), indicating that progestogen type has an impact on the ability to handle an increasing working memory load. No significant differences in WMI or RM errors were detected for this comparison in the Asymptotic Phase.

**FIGURE 5:**
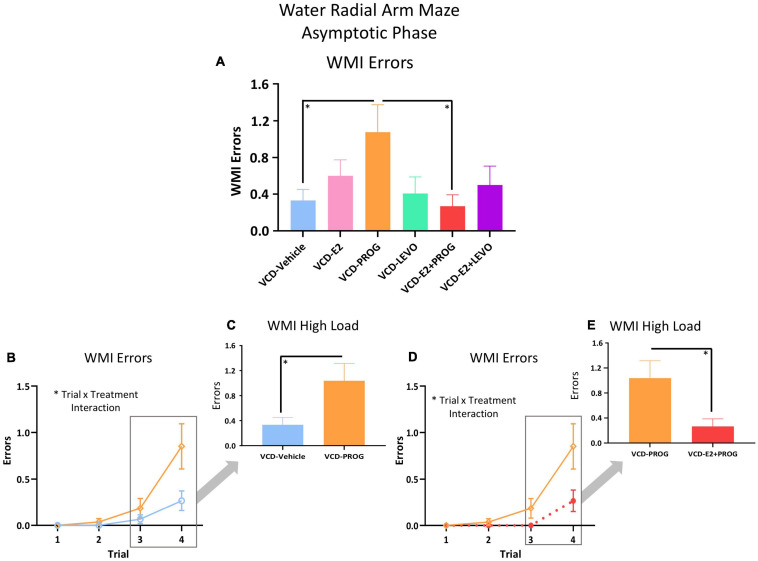
WMI Errors During the Asymptotic Phase. **(A)** Across all trials, a main effect of Treatment was present between the VCD-PROG group and the VCD-Vehicle group (*p* < 0.05) as well as compared to the VCD-E2 + PROG group (*p* < 0.05). **(B)** VCD-PROG vs. VCD-Vehicle comparison: A Trial × Treatment interaction was present for this comparison (*p* < 0.05) **(C)** When High Load trials (Trials 3 + 4) were assessed, VCD-PROG rats made more WMI errors than VCD-Vehicle rats (*p* < 0.05). **(D)** VCD-PROG vs. VCD-E2 + PROG comparison: A Trial × Treatment interaction was present for this comparison (*p* < 0.05). **(E)** When High Load trials (Trials 3 + 4) were assessed, VCD-PROG rats made more WMC errors than VCD-E2 + PROG rats (*p* < 0.05). Significance: ^∗^ = *p* < 0.05.

For WMI, there was a main effect of Treatment [*F*_(__1_,_17__)_ = 5.26, *p* < 0.05; Figure 5A] and a Trial × Treatment interaction [*F*_(__3_,_51__)_ = 2.87, *p* < 0.05; Figure 5B] whereby follicle-deplete rats treated with progesterone made more WMI errors compared to those without subsequent hormone treatment. When Trial 3 + Trial 4, the highest working memory load trials, were evaluated for WMI errors, there was a main effect of Treatment [*F*_(__1_,_17__)_ = 5.21, *p* < 0.05; Figure 5C], again indicating that follicle-deplete rats treated with progesterone made more WMI errors when working memory load was burdened compared to transitionally menopausal rats that did not receive subsequent hormone treatment. No differences between WMC or RM errors were present for this comparison.

##### What role does daily combination hormone therapy play for spatial learning and memory with transitional menopause?

**FIGURE 6:**
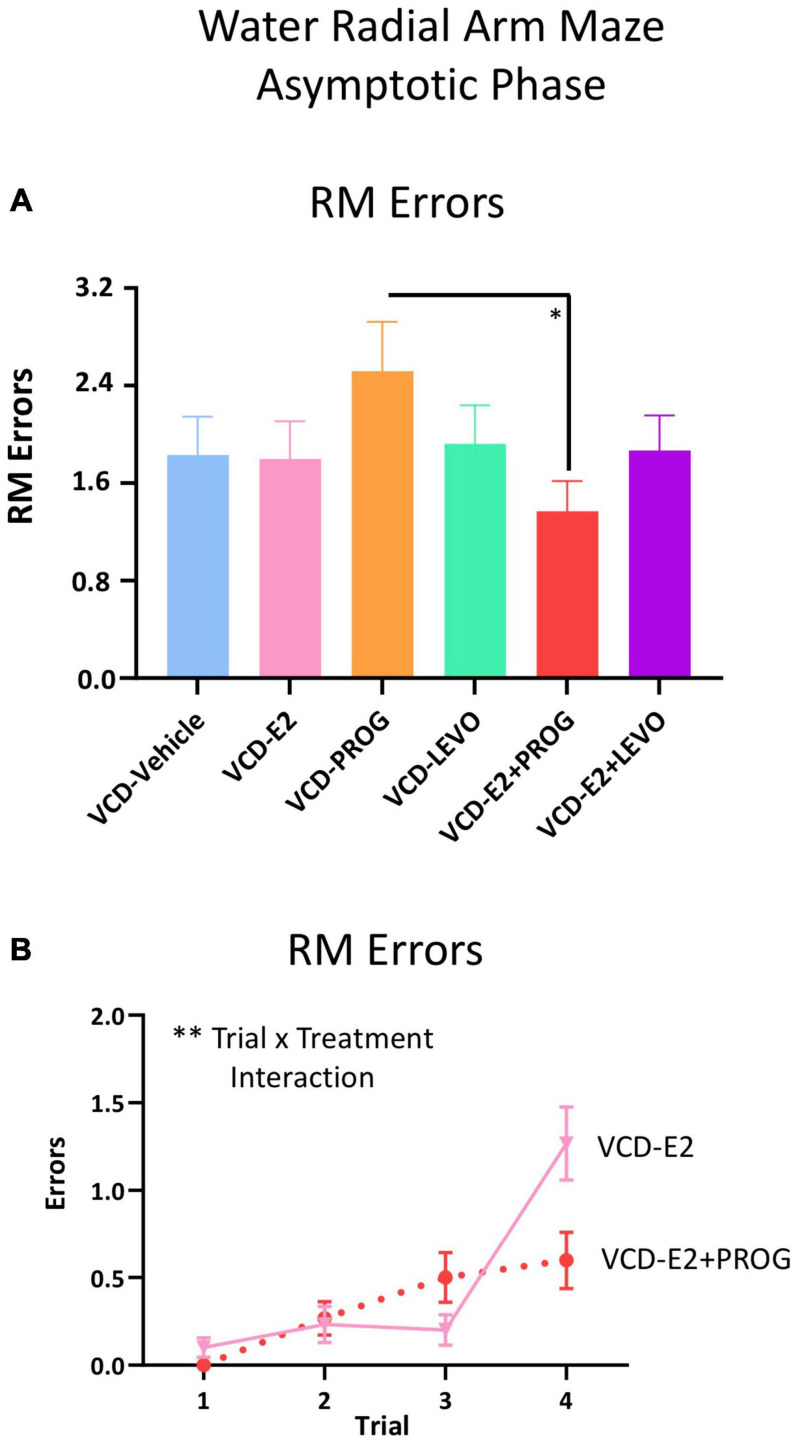
RM Errors During the Asymptotic Phase. **(A)** Across all trials, a main effect of Treatment was present between the VCD-PROG group and the VCD-E2 + PROG group (*p* < 0.05). **(B)** VCD-E2 vs. VCD-E2 + PROG comparison: A Trial × Treatment interaction occurred (*p* < 0.01). Significance: ^∗^ = *p* < 0.05, ^∗∗^ = *p* < 0.01.

During the Asymptotic Phase of testing, there was a Trial × Treatment interaction for WMC errors within the VCD-PROG group vs. VCD-E2 + PROG group comparison [*F*_(__2_,_34__)_ = 3.42, *p* < 0.05; Figure 4B]; when the Trial 3 + Trial 4, the high working memory load trials, were probed for this comparison, there was a main effect of Treatment for WMC errors[*F*_(__1_,_17__)_ = 4.66, *p* < 0.05; Figure 4C], where rats treated with E2 plus progesterone made fewer errors than progesterone-only counterparts. Similarly, for WMI errors, there was a main effect of Treatment for the VCD-PROG vs. VCD-E2 + PROG comparison [*F*_(__1_,_17__)_ = 6.64, *p* < 0.05; Figure 5A], indicating that the addition of E2 to progesterone treatment enhanced performance compared to progesterone alone on WMI errors across all trials; a Trial × Treatment interaction [*F*_(__3_,_51__)_ = 3.17, *p* < 0.05; Figure 5D] was also present for this comparison. When Trial 3 + Trial 4, the high working memory load trials, were probed for WMI errors, a main effect of Treatment persisted [*F*_(__1_,_17__)_ = 6.67, *p* < 0.05; Figure 5E], where combined E2 plus progesterone treatment enhanced performance compared to progesterone-only treatment, particularly when memory load was highly burdened for WMI errors. A main effect of Treatment was also present for RM errors between VCD-PROG and VCD-E2 + PROG groups [*F*_(__1_,_17__)_ = 7.56, *p* < 0.05; Figure 6A]. As such, across all error types, a daily combination treatment of E2 plus progesterone treatment enhanced spatial memory performance compared to progesterone-only treatment in transitionally menopausal rats in the Asymptotic Phase. When E2-only treatment was compared to this combination of daily E2 plus progesterone, a Trial × Treatment interaction for RM errors was present [*F*_(__3_,_54__)_ = 5.72, *p* < 0.01; Figure 6B] with a higher mean error score for the VCD-E2 treated group as compared to the combined VCD-E2+PROG treated group on Trial 4, suggesting a potential benefit for the VCD-E2 + PROG group’s spatial reference memory at the highest working memory load compared to E2-only treatment as well, although RM performance across trials should be interpreted with caution given a cap of four possible RM errors. Collectively, when ovaries remained structurally intact but were follicle-deplete, combined E2 plus progesterone treatment improved spatial memory performance compared to treatment with E2 alone or progesterone alone.

#### Six-Hour Delay

**FIGURE 7:**
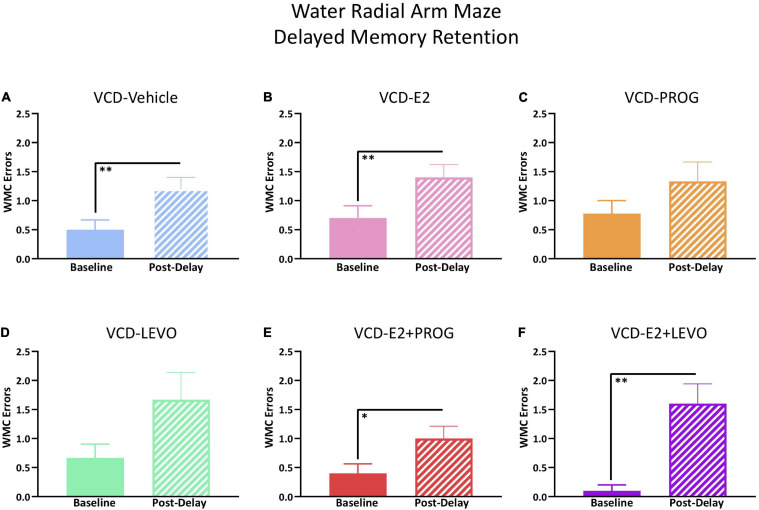
WRAM Six-Hour Delayed Memory Retention Test. **(A)** The VCD-Vehicle group exhibited a delay-induced working memory impairment compared to the previous day’s baseline performance (*p* < 0.01). **(B)** The VCD-E2 group exhibited a delay-induced working memory impairment compared to the previous day’s baseline performance (*p* < 0.01). **(C)** The VCD-PROG group did not display a delay-induced working memory impairment compared to the previous day’s baseline performance. **(D)** The VCD-LEVO group did not display a delay-induced working memory impairment compared to the previous day’s baseline performance. **(E)** The VCD-E2 + PROG group exhibited a delay-induced working memory impairment compared to the previous day’s baseline performance (*p* < 0.05). **(F)** The VCD-E2 + LEVO group exhibited a delay-induced working memory impairment compared to the previous day’s baseline performance (*p* < 0.01). Significance: ^∗^ = *p* < 0.05, ^∗∗^ = *p* < 0.01.

Treatment groups were analyzed separately for delayed memory retention assessment. WMC errors committed on the first post-delay trial (Trial 3) were compared to errors on Trial 3 on the last day of baseline testing. There was a main effect of Delay Day for the VCD-Vehicle group [*F*_(__9_,_1__)_ = 10.76, *p* < 0.01; Figure 7A], VCD-E2 group [*F*_(__9_,_1__)_ = 21.00, *p* < 0.01; Figure 7B], VCD-E2 + PROG group [*F*_(__9_,_1__)_ = 7.36, *p* < 0.05; Figure 7E], and VCD-E2 + LEVO group [*F*_(__9_,_1__)_ = 19.29, *p* < 0.01; Figure 7F], where most groups made more errors when an extended delay occurred, regardless of hormone therapy regimen. Analyses did not reach statistical significance for the VCD-PROG (Figure 7C) or VCD-LEVO group (Figure 7D), suggesting that the progestogen-only treatments promoted some level of memory retention across the delay period.

### Morris Water Maze

**FIGURE 8:**
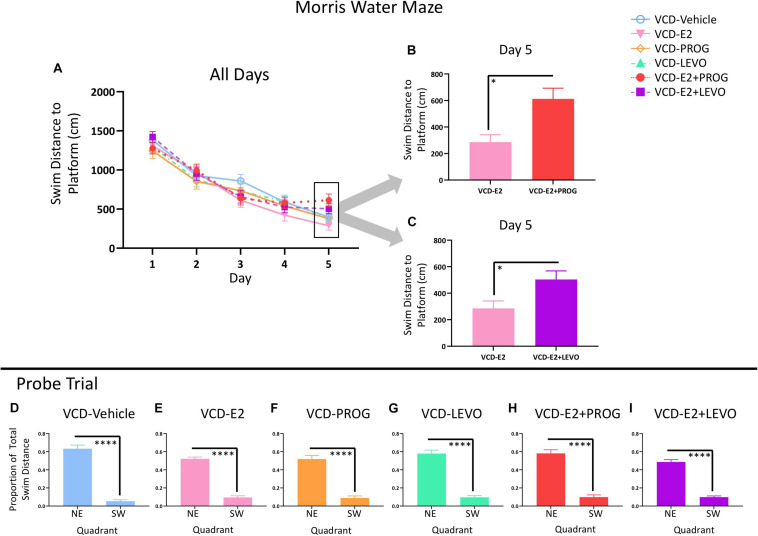
Morris Water Maze. **(A)** Swim Distance to Platform Across All Days. **(B)** VCD-E2 treated rats swam less distance to the platform compared to the VCD-E2 + PROG treated rats (*p* < 0.05). **(C)** VCD-E2 treated rats swam less distance to the platform compared to the VCD-E2 + LEVO treated rats (*p* < 0.05). **(D–I)** Probe trial. All treatment groups swam a greater proportion of total distance in the previously platformed quadrant vs. the opposite quadrant, indicating that all groups spatially localized to the hidden platform location. Significance: ^∗^ = *p* < 0.05, ^****^p < 0.0001.

Figure 8A demonstrates MM performance across the 5-day task.

#### What Role Does Daily E2-Only Treatment Have on a Simple Spatial Reference Memory Task With Transitional Menopause?

There were no Treatment effects across all 5 days of the task or on Day 5 alone between VCD-Vehicle and VCD-E2 groups, indicating that daily E2 treatment at the given dose did not alter spatial reference memory compared to follicle-deplete rats that did not receive subsequent hormone treatment.

#### Does Daily Treatment With an Individual Progestogen Impact Cognition With Transitional Menopause, and Is Type of Progestogen a Factor for a Simple Spatial Reference Memory Task?

There were no Treatment effects for any planned comparison including the progestogen-only groups across all 5 days of the task or on Day 5 alone.

#### What Role Does Daily Combination Hormone Therapy Play for a Simple Spatial Reference Memory Task With Transitional Menopause?

The VCD-E2 vs. VCD-E2 + PROG comparison yielded a Trial × Treatment interaction across all days of MM testing [*F*_(__3_,_216__)_ = 2.78, *p* < 0.05]. On the final testing day, there was a main effect of Treatment for the VCD-E2 vs. VCD-E2 + PROG comparison [*F*_(__1_,_18__)_ = 7.59, *p* < 0.05; Figure 8B] and the VCD-E2 vs. VCD-E2 + LEVO comparison [*F*_(__1_,_18__)_ = 5.22, *p* < 0.05; Figure 8C], where follicle-deplete rats treated with only E2 swam less distance to the platform compared to follicle-deplete rats administered a combination hormone therapy treatment. Thus, the addition of an exogenous progestogen, whether it was an endogenous-like progesterone or the synthetic progestin levonorgestrel, in combination with E2 impaired performance compared to E2 administration alone at the end of this simple spatial reference memory task.

#### Probe Trial

Probe trial analysis demonstrated that each treatment group effectively learned to use a spatial strategy to solve the MM task (Figures 8D–I). Indeed, when the platform was removed from the maze, each treatment group spent a greater proportion of total swim distance in the previously platformed target quadrant compared to the opposite quadrant (VCD-Vehicle: [*F*_(__9_,_1__)_ = 150.44, *p* < 0.0001]; VCD-E2: [*F*_(__9_,_1__)_ = 159.271, *p* < 0.0001]; VCD-PROG: [*F*_(__8_,_1__)_ = 52.40, *p* < 0.0001]; VCD-LEVO: [*F*_(__8_,_1__)_ = 82.03, *p* < 0.0001]; VCD-E2 + PROG: [*F*_(__9_,_1__)_ = 66.32, *p* < 0.0001]; VCD-E2 + LEVO: [*F*_(__9_,_1__)_ = 159.306, *p* < 0.0001]).

### Visible Platform

**FIGURE 9:**
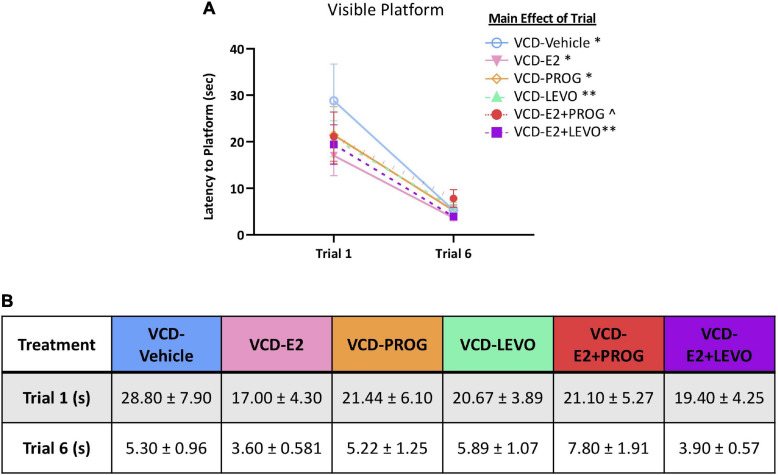
Visible Platform. **(A)** All subjects decreased latency to platform from the first to last trial. **(B)** Trial times (means + S.E.M.) for each treatment group are provided. Significance: ^∗^ = *p* < 0.05, ^∗∗^ = *p* < 0.01, ^ = *p* = 0.06.

When comparing performance on the first trial vs. the last trial for each treatment group, there was a main effect of Trial for the VCD-Vehicle group [*F*_(__9_,_1__)_ = 9.16, *p* < 0.05], VCD-E2 group [*F*_(__9_,_1__)_ = 8.88, *p* < 0.05], VCD-PROG group [*F*_(__8_,_1__)_ = 6.59, *p* < 0.05], VCD-LEVO group [*F*_(__8_,_1__)_ = 15.67, *p* < 0.01], and VCD-E2 + LEVO group [*F*_(__9_,_1__)_ = 15.62, *p* < 0.01]. The VCD-E2 + PROG group Trial effect was marginal [*F*_(__9_,_1__)_ = 4.50, *p* = 0.06], although this was likely due to one subject in that group that took 22 s to reach the platform on Trial 6 (Figure 9A). When this subject was excluded from the analysis, the Trial effect became significant [*F*_(__8_,_1__)_ = 7.08, *p* < 0.05]. However, all groups, including the VCD-E2 + PROG group, decreased in average trial latency from Trial 1 to Trial 6 of the VP task, with an average latency to platform of 5.3 ± 0.49 s on Trial 6 (Figure 9B).

### Open Field Task

**FIGURE 10:**
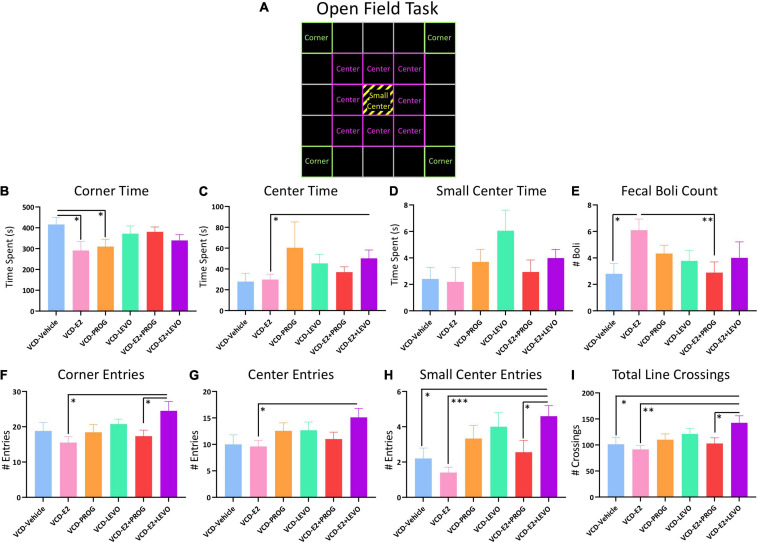
Open Field Task. **(A)** Schematic of the OFT arena. Green squares indicate which boxes were defined as Corners, pink squares indicate which boxes were defined as Center, and yellow stripes indicate the Small Center, which was also included in the “Center” measure. **(B)** The VCD-Vehicle group spent more time in the corners compared to VCD-E2 and VCD-PROG groups. **(C)** VCD-E2 + LEVO treatment increased time spent in the center compared to VCD-E2 treatment. **(D)** No significant differences in Small Center Time were detected. **(E)** The VCD-E2 group had more fecal boli than the VCD-Vehicle and VCD-E2 + PROG group. **(F)** The VCD-E2 + LEVO group made more entries into the corner compared to the VCD-E2 group as well as the VCD-E2 + PROG group. **(G)** The VCD-E2 + LEVO group made more entries into the center compared to VCD-E2 treatment alone. **(H)** The VCD-E2 + LEVO group made more entries into the small center compared to VCD-Vehicle group, VCD-E2 group, and VCD-E2 + PROG group. **(I)** Total Line Crossing analyses indicate that the VCD-E2 + LEVO group moved more in the OFT compared to VCD-Vehicle, VCD-E2, and VCD-E2+PROG groups. Significance: ^∗^ = *p* < 0.05, ^∗∗^ = *p* < 0.01, ^∗∗∗^ = *p* < 0.001.

One subject from the VCD-E2 + PROG group was excluded from OFT analyses due to a technical error. Figure 10A provides a schematic of the OFT with boxes overlaid to operationally define the Corners, Center, and Small Center within the arena.

#### What Role Does Daily E2-Only Treatment Have in Anxiety-Like Behaviors With Transitional Menopause?

Transitionally menopausal rats treated with daily E2-only spent less time in the corners of the OFT when compared to transitionally menopausal rats treated with no hormone [Treatment main effect for the VCD-Vehicle vs. VCD-E2 comparison: *F*_(__1_,_18__)_ = 5.24, *p* < 0.05], suggesting decreased anxiety-like behavior when E2-only hormone therapy is given after follicular depletion as compared to no hormone therapy given after follicular depletion (Figure 10B). There were no effects present for time in the Center or Small Center for this comparison, nor were there differences in entries into the Corners, Center, or Small Center.

#### Does Daily Treatment With an Individual Progestogen Impact Anxiety-Like Behavior With Transitional Menopause, and Is Type of Progestogen a Factor for Outcomes?

Regarding Corner Time (s), transitionally menopausal rats treated with daily progesterone alone spent less time in the corners of the OFT when compared to counterparts without hormone treatment [Treatment main effect VCD-Vehicle vs. VCD-PROG comparison: *F*_(__1_,_17__)_ = 4.80, *p* < 0.05], suggesting a decrease in anxiety-like behavior for the progesterone-treated group (Figure 10B). There were no effects present for time in the Center or Small Center for these comparisons, nor were there differences in entries into the Corners, Center, or Small Center.

#### What Role Does Daily Combination Hormone Therapy Play for Anxiety-Like Behavior With Transitional Menopause?

Analysis of Center Time (s) revealed a Treatment effect for the VCD-E2 vs. VCD-E2 + LEVO comparison [*F*_(__1_,_18__)_ = 4.61, *p* < 0.05], wherein subjects treated with a combination of E2 and levonorgestrel spent significantly more time in the Center of the open field, indicating reduced anxiety-like behavior, compared to rats treated with E2-only (Figure 10C). There were no other effects present for Corner time or Small Center time for these comparisons. When assessing entries into the Corners, there were Treatment effects for the VCD-E2 vs. VCD-E2 + LEVO comparison [*F*_(__1_,_18__)_ = 8.20, *p* < 0.05] and VCD-E2 + PROG vs. VCD-E2 + LEVO comparison [*F*_(__1_,_17__)_ = 4.87, *p* < 0.05]. In both analyses, the VCD-E2 + LEVO group showed increased entries into the corners (Figure 10F). A Treatment effect was also indicated within the VCD-E2 vs. VCD-E2 + LEVO comparison for Center entries [*F*_(__1_,_18__)_ = 7.14, *p* < 0.05] (Figure 10G) and Small Center entries [*F*_(__1_,_18__)_ = 22.59, *p* < 0.001] (Figure 10H). Increased Small Center entries were also evident in the VCD-E2 + LEVO group compared to the VCD-Vehicle group [*F*_(__1_,_18__)_ = 8.10, *p* < 0.05] and the VCD-E2 + PROG [*F*_(__1_,_17__)_ = 5.21, *p* < 0.05] (Figure 10H).

#### Line Crossings Analyses

Total Line Crossings, measuring total locomotion, differed for VCD-Vehicle vs. VCD-E2 + LEVO groups [*F*_(__1_,_18__)_ = 4.64, *p* < 0.05], VCD-E2 vs. VCD-E2 + LEVO groups [*F*_(__1_,_18__)_ = 10.81, *p* < 0.01], and VCD-E2 + PROG vs. VCD-E2 + LEVO groups [*F*_(__1_,_17__)_ = 5.11, *p* < 0.05], with rats treated with a combination of daily E2 plus levonorgestrel exhibiting increased locomotor activity in the OFT overall (Figure 10I). Transitionally menopausal rats treated with E2-only produced more fecal boli compared to rats without hormone therapy treatment (VCD-Vehicle vs. VCD-E2: *F*_(__1_,_18__)_ = 8.27, *p* < 0.05) and compared to rats treated with a combination of E2 plus progesterone (VCD-E2 vs. VCD-E2 + PROG: [*F*_(__1_,_18__)_ = 8.87, *p* < 0.01]) during the 10 min trial (Figure 10E).

### Forced Swim Task

#### What Role Does Daily E2-Only Treatment Have in Depressive-Like Behaviors With Transitional Menopause?

**FIGURE 11:**
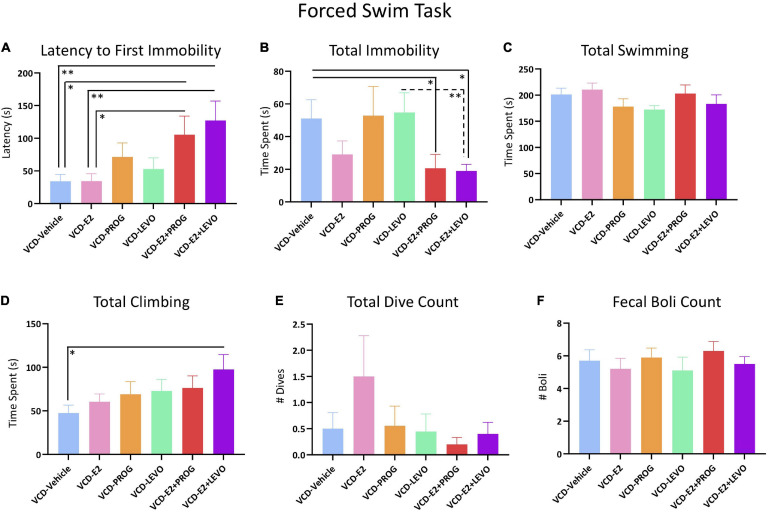
Forced Swim Test. **(A)** Both combination hormone therapy groups had a longer latency to immobility when compared to VCD-Vehicle or VCD-E2 groups, suggesting an antidepressant-like effect of combination hormone therapy compared to no treatment or E2 treatment alone. **(B)** Total immobility was decreased in the combination hormone therapy groups, again suggesting an antidepressant-like effect compared to Vehicle treatment or LEVO-alone treatment. **(C)** No Treatment differences were indicated in time spent swimming. **(D)** The VCD-E2 + LEVO group spent more time climbing compared to the VCD-Vehicle group, indicating antidepressant-like effects. **(E,F)** Total Dive Count and Fecal Boli Counts did not differ among treatment comparisons. Significance: ^∗^ = *p* < 0.05, ^∗∗^ = *p* < 0.01.

Latency to Immobility, Total Immobility Duration, Total Swimming Duration, Total Climbing Duration, Number of Dives, or Number of Fecal Boli did not differ between rats treated with E2 only compared to counterparts not administered subsequent hormone treatment (Figure 11).

#### Does Daily Treatment With an Individual Progestogen Impact Depressive-Like Behavior With Transitional Menopause, and Does Type of Progestogen Have an Impact?

No differences were found in Latency to Immobility, Total Immobility Duration, Total Swimming Duration, Total Climbing Duration, Number of Dives, or Number of Boli for any planned comparison including the VCD-Vehicle group compared to the VCD-PROG or VCD-LEVO group, nor did VCD-PROG and VCD-LEVO groups differ from one another (Figure 11).

#### What Role Does Daily Combination Hormone Therapy Play for Depressive-Like Behavior With Transitional Menopause?

Regarding Latency to Immobility, there was a Treatment effect for the VCD-Vehicle vs. VCD-E2 + PROG comparison [*F*_(__1_,_18__)_ = 5.51, *p* < 0.05], the VCD-Vehicle vs. VCD-E2 + LEVO comparison [*F*_(__1_,_18__)_ = 8.63, *p* < 0.01], the VCD-E2 vs. VCD-E2 + PROG comparison [*F*_(__1_,_18__)_ = 5.35, *p* < 0.05], and the VCD-E2 vs. VCD-E2 + LEVO comparison [*F*_(__1_,_18__)_ = 8.42, *p* < 0.01]. In all comparisons, transitionally menopausal rats treated with combined E2 plus progestogen hormone treatment regimens had longer latencies to immobility, indicating that the addition of either natural progesterone or the synthetic progestin levonorgestrel to E2 treatment yields antidepressant-like behavior compared to E2-only treatment or no hormone treatment following transitional menopause (Figure 11A). Furthermore, Total Immobility Duration was increased in the VCD-Vehicle group compared to the VCD-E2 + PROG group [*F*_(__1_,_18__)_ = 4.55, *p* < 0.05], and compared to the VCD-E2 + LEVO group [*F*_(__1_,_18__)_ = 6.94, *p* < 0.05]. In both comparisons, the groups treated with combined E2 plus progestogen hormone regimens spent less total time immobile, indicating that combined hormone therapy regimens induce antidepressant-like behavior compared to no hormone treatment with transitional menopause (Figure 11B). Additionally, VCD-LEVO vs. VCD-E2 + LEVO differed for Total Immobility Duration [*F*_(__1_,_17__)_ = 8.65, *p* < 0.01], where rats treated with levonorgestrel alone spent more time immobile compared to counterparts treated with a combination of E2 plus levonorgestrel (Figure 11B). Although Total Swimming Duration did not differ for any comparison (Figure 11C), rats treated with a combination of E2 plus levonorgestrel spent more time presenting with climbing behavior compared to counterparts that did not receive hormone therapy after follicular depletion (VCD-Vehicle vs. VCD-E2 + LEVO: [*F*_(__1_,_18__)_ = 6.62, *p* < 0.05]) (Figure 11D). Taken together, these results suggest that a combined hormone therapy regimen, particularly a combination of E2 and levonorgestrel, results in antidepressant-like effects compared to no hormone treatment, E2-only treatment, or progestogen-only treatment after transitional menopause.

### Vaginal Cytology

Across two consecutive days of vaginal cytology monitoring, most VCD-Vehicle-treated rats exhibited mixed cytology resembling metestrus-like smears, suggesting disrupted estrous cyclicity, which is expected following accelerated follicular depletion without subsequent hormone therapy treatment. Rats that received E2 only displayed primarily cornified cells resembling estrus-like smears, which was expected as a result of daily E2 administration. Rats treated with progesterone only or levonorgestrel only had primarily metestrus- or diestrus- like smears, indicative of a relatively higher ratio of circulating progesterone to estrogen levels. The VCD-E2 + PROG group presented with cytology mostly resembling metestrus-like smears, and some diestrus-like smears, while the VCD-E2 + LEVO group showed estrus- and metestrus- like smears. Based on prior data from our and other laboratories, normal estrous cyclicity is disrupted approximately 4 months after VCD injection administration, and vaginal cytology can be modified by a given hormone therapy regimen (Koebele et al., 2020a).

### Serum Hormone Levels

**FIGURE 12:**
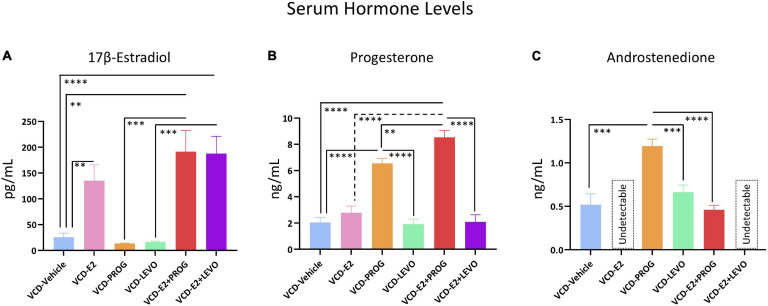
Serum Hormone Levels. **(A)** E2 was elevated in VCD-E2, VCD-E2 + PROG, and VCD-E2 + LEVO groups compared to VCD-Vehicle rats. Additionally, combination hormone therapy groups had elevated E2 compared to their respective progestogen-only groups. E2 analysis n/group: VCD-Vehicle *n* = 9; VCD-E2 *n* = 10; VCD-PROG *n* = 9; VCD-LEVO *n* = 9; VCD-E2 + PROG *n* = 10; VCD-E2 + LEVO *n* = 10. **(B)** Progesterone was elevated in the VCD-PROG group and the VCD-E2 + PROG group compared to the VCD-Vehicle group, VCD-E2 group, and VCD-LEVO group. The combination hormone group had higher progesterone levels compared to the VCD-PROG group alone. Progesterone analysis n/group: VCD-Vehicle *n* = 10; VCD-E2 *n* = 10; VCD-PROG *n* = 9; VCD-LEVO *n* = 9; VCD-E2 + PROG *n* = 10; VCD-E2 + LEVO *n* = 10. **(C)** All subjects in the VCD-E2 group and VCD-E2 + LEVO group had undetectable levels of androstenedione. Androstenedione was elevated in the VCD-PROG group compared to VCD-Vehicle, VCD-LEVO, and VCD-E2 + PROG groups. Androstenedione analysis n/group: VCD-Vehicle *n* = 9; VCD-E2 *n* = 0 [undetectable]; VCD-PROG *n* = 9; VCD-LEVO *n* = 9; VCD-E2 + PROG *n* = 10; VCD-E2 + LEVO *n* = 0 [undetectable]. Significance: ^∗∗^ = *p* < 0.01, ^∗∗∗^ = *p* < 0.001, ^****^ = *p* < 0.0001.

One VCD-Vehicle rat, all VCD-E2, and all VCD-E2 + LEVO rats were excluded from the androstenedione analyses because the measured serum hormone level was below the detectable limit of the assay. Additionally, one VCD-Vehicle rat was excluded from the E2 analyses due to insufficient serum volume needed to run the assay. The n per group for each steroid hormone assay is included in the Figure 12 caption summarizing serum hormone levels.

#### How Does Daily E2-Only Treatment Affect Serum Hormone Profiles With Transitional Menopause?

Transitionally menopausal rats treated with daily E2 had increased circulating E2 levels compared to the Vehicle-treated counterparts, as expected [*F*_(__1_,_17__)_ = 10.82, *p* < 0.01] (Figure 12A). Progesterone levels did not differ between VCD-Vehicle and VCD-E2 groups (Figure 12B). Lastly, all subjects within the VCD-E2 group had undetectable levels of androstenedione, and thus the comparison could not be carried out between VCD-Vehicle vs. VCD-E2 groups (Figure 12C).

#### How Does Daily Treatment With Progesterone or Levonorgestrel Affect Serum Hormone Levels With Transitional Menopause, and Does Type of Progestogen Impact Outcomes?

Treatment with progesterone or levonorgestrel did not alter circulating E2 levels compared to transitionally menopausal counterparts that did not receive hormone treatment or compared to each other (Figure 12A). The VCD-PROG group had higher circulating progesterone levels than the VCD-Vehicle group [*F*_(__1_,_17__)_ = 70.95, *p* < 0.0001] and the VCD-LEVO group [*F*_(__1_,_16__)_ = 71.26, *p* < 0.0001] (Figure 12B). Rats treated with levonorgestrel had similar circulating progesterone profiles compared to transitionally menopausal rats that did not receive hormone therapy, suggesting that this synthetic progestin did not alter endogenous progesterone levels in follicle-deplete ovary-intact rats. Interestingly, the VCD-PROG group had higher androstenedione levels compared to the VCD-Vehicle group [*F*_(__1_,_16__)_ = 20.53, *p* < 0.001], and compared to the VCD-LEVO group [*F*_(__1_,_16__)_ = 21.49, *p* < 0.001] (Figure 12C), suggesting that follicle-deplete rats with exogenous administration of natural progesterone experience increased circulating androgen levels compared to follicle-deplete rats without hormone treatment, or compared to those treated with the synthetic progestin levonorgestrel. On the other hand, treatment with levonorgestrel alone did not impact circulating androstenedione levels compared to counterparts that did not receive hormone therapy.

#### How Does Daily Combination Hormone Therapy Affect Serum Hormone Levels With Transitional Menopause?

Compared to rats without hormone treatment, rats in both combined hormone therapy groups demonstrated increased levels of circulating E2 (VCD-Vehicle vs. VCD-E2 + PROG group [*F*_(__1_,_17__)_ = 14.18, *p* < 0.01]; VCD-Vehicle vs. VCD-E2 + LEVO [*F*_(__1_,_17__)_ = 20.21, *p* < 0.0001]). Circulating E2 did not differ between VCD-E2 and VCD-E2 + PROG groups or VCD-E2 and VCD-E2 + LEVO groups, indicating that the addition of a progestogen to E2 treatment was insufficient to alter circulating E2 levels, at least at the given doses. Likewise, rats treated with either type of progestogen independently had less circulating E2 compared to their respective combined hormone therapy group (VCD-PROG vs. VCD-E2 + PROG [*F*_(__1_,_17__)_ = 16.83, *p* < 0.001]; VCD-LEVO vs. VCD-E2 + LEVO [*F*_(__1_,_17__)_ = 23.44, *p* < 0.001]). The VCD-E2 + PROG vs. VCD-E2 + LEVO groups did not differ in circulating E2 levels; thus, the type of progestogen (i.e., natural progesterone or synthetic progestin levonorgestrel) did not impact circulating E2 levels when the hormone therapy was administered in a combined estrogen plus progestogen fashion. Overall, the E2 component is likely the primary driver in determining circulating E2 levels in a given group (Figure 12A).

The VCD-E2 + PROG group had increased circulating progesterone levels compared to the VCD-Vehicle group [*F*_(__1_,_18__)_ = 103.78, *p* < 0.0001], the VCD-E2 group [*F*_(__1_,_18__)_ = 62.29, *p* < 0.0001], the VCD-E2 + LEVO group [*F*_(__1_,_18__)_ = 74.99, *p* < 0.0001], and, interestingly, the VCD-PROG alone group [*F*_(__1_,_17__)_ = 9.36, *p* < 0.01]; the outcome from this latter comparison indicates that combined E2 plus progesterone therapy may have a synergistic effect on increasing circulating progesterone levels compared to progesterone-only treatment. Circulating progesterone levels did not differ between VCD-Vehicle vs. VCD-E2 + LEVO groups, VCD-E2 vs. VCD-E2 + LEVO groups, or VCD-LEVO vs. VCD-E2 + LEVO groups, suggesting that the synthetic progestin levonorgestrel does not influence endogenous progesterone production itself, at least at the dose given in this experiment (Figure 12B).

All subjects in the VCD-E2 + LEVO group had undetectable levels of circulating androstenedione, and thus could not be evaluated relative to respective comparison groups. Because all subjects treated with E2 only likewise had undetectable androstenedione levels, this group also could not be compared to the VCD-E2 + PROG group. The VCD-E2 + PROG group did not differ in androstenedione levels from the VCD-Vehicle group. Androstenedione levels differed between VCD-PROG and VCD-E2 + PROG groups, whereby the combination hormone therapy regimen yielded reduced androstenedione levels compared to progesterone treatment alone [*F*_(__1_,_17__)_ = 62.90, *p* < 0.0001] (Figure 12C).

### Ovarian Follicle Counts

Two subjects from the VCD-Vehicle group, two subjects from the VCD-LEVO group, one subject from the VCD-E2 + PROG group, and one subject from the VCD-E2 + LEVO group were excluded from follicle analyses due to poor tissue quality. Thus, the n/group for all follicle analyses was the following: VCD-Vehicle *n* = 8, VCD-E2 *n* = 10, VCD-PROG *n* = 9, VCD-LEVO *n* = 7, VCD-E2 + PROG *n* = 9, and VCD-E2 + LEVO = 9. The independent ovary-intact Vehicle reference group *n* = 10.

#### How Does Daily E2-Only Treatment Affect Ovarian Follicle Profiles With Transitional Menopause?

**FIGURE 13:**
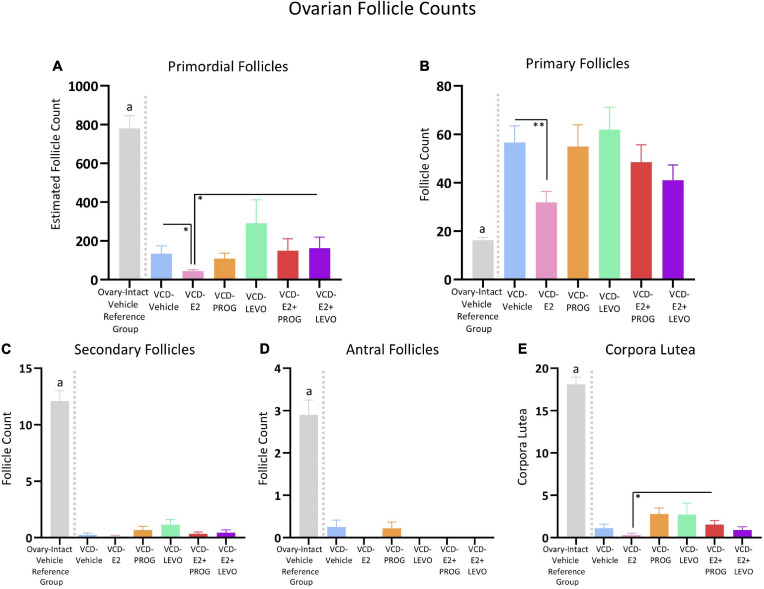
Ovarian Follicle Counts. An independent ovary-intact reference group (*n* = 10) is included to assess successful follicular depletion following VCD treatment. The letter “a” indicates that this ovary-intact reference group was significantly different from each VCD-treated group. **(A)** Estimated primordial follicle counts were decreased in the VCD-E2 group compared to the VCD-Vehicle group and the VCD-E2 + LEVO group. **(B)** Primary follicles were decreased in the VCD-E2 group compared to the VCD-Vehicle group, replicating prior work. **(C)** Secondary follicle counts were significantly depleted in VCD-treated groups, indicating successful accelerated follicular atresia. **(D)** Antral follicle counts were significantly depleted in VCD-treated groups, indicating successful accelerated follicular atresia. **(E)** The VCD-E2 + PROG group had more corpora lutea compared to the VCD-E2 group, suggesting occasional ovulatory cycles in this group during the transition to reproductive senescence. Significance: ^∗^ = *p* < 0.05, ^∗∗^ = *p* < 0.01.

Compared to the VCD-Vehicle group, the VCD-E2 group had significantly fewer primordial follicles [*F*_(__1_,_16__)_ = 6.10, *p* < 0.05] and fewer primary follicles [*F*_(__1_,_16__)_ = 9.89, *p* < 0.01] (Figures 13A,B), an effect we have previously observed in follicle-depleted rats with tonic E2 treatment (Koebele et al., 2020a). Secondary follicles, antral follicles, and corpora lutea counts did not differ between VCD-Vehicle and VCD-E2 groups, although both groups exhibited substantial follicle decline, indicating successful VCD-induced follicular depletion. In fact, there were no detectable antral follicles for any subject treated with E2 only (Figures 13C–E).

#### How Does Daily Treatment With Progesterone or Levonorgestrel Affect Ovarian Follicle Profiles With Transitional Menopause, and Does Type of Progestogen Matter?

There were no Treatment group differences in primordial follicles, primary follicles, secondary follicles, antral follicles, or corpora lutea counts in the VCD-Vehicle group vs. the VCD-PROG group or vs. VCD-LEVO group, nor did the VCD-PROG and VCD-LEVO groups differ from each other, indicating that progestogen treatment alone does not impact the composition of the ovarian follicle pool in an accelerated follicular depletion model (Figures 13A–E).

#### How Does Daily Combination Hormone Therapy Affect Ovarian Follicle Profiles in a Model of Transitional Menopause?

Estimated primordial follicle counts did not differ for VCD-Vehicle rats compared to the VCD-E2 + PROG group or compared to the VCD-E2 + LEVO group. Compared to transitionally menopausal rats treated with E2 only, transitionally menopausal rats treated with E2 plus levonorgestrel had more primordial follicles [*F*_(__1_,_17__)_ = 4.86, *p* < 0.05] (Figure 13A), suggesting that this combined hormone treatment protects remaining healthy follicles in the ovarian reserve during this menopause transition time point compared to treatment with E2 alone. Estimated primordial follicle counts, primary follicles, secondary follicles, and antral follicles did not differ for combined hormone therapy groups compared to their respective progestogen counterparts, nor did they differ from each other. In addition, the VCD-E2 + PROG group had more corpora lutea compared to the VCD-E2 group [*F*_(__1_,_17__)_ = 6.93, *p* < 0.05], indicating that rats treated with E2 plus progesterone may have occasional ovulatory cycles during the menopause transition, although both groups were all significantly depleted and categorized as infertile (Figure 13E).

#### Confirmation of Follicular Depletion in VCD-Treated Groups: Comparison to an Ovary-Intact Vehicle Reference Group

Overall, groups treated with VCD showed substantial ovarian follicle loss in comparison to normally aging ovary-intact rats that did not receive exposure to VCD. To confirm that VCD treatment depleted the ovarian follicle reserve in all treatment groups in the current study, we utilized an independent data set of ovarian follicle counts collected in our laboratory from rats that received the complementary Vehicle injection for VCD administration, similar to a comparison procedure we have published previously (Koebele et al., 2020a). This ovary-intact Vehicle reference group was compared to each VCD-treated group in the current study (Figures 13A–E; specific comparisons below), with analyses showing that each VCD group had fewer primordial follicles, secondary follicles, antral follicles, and corpora lutea than this ovary-intact Vehicle reference group.

For primordial follicles, there was a Treatment main effect for each group comparison with the ovary-intact Vehicle reference group: VCD-Vehicle: [*F*_(__1_,_16__)_ = 62.55, *p* < 0.0001]; VCD-E2: [*F*_(__1_,_18__)_ = 125.72, *p* < 0.0001]; VCD-PROG: [*F*_(__1_,_17__)_ = 82.70, *p* < 0.0001]; VCD-LEVO: [*F*_(__1_,_15__)_ = 14.79, *p* < 0.01]; VCD-E2 + PROG: [*F*_(__1_,_17__)_ = 48.98, *p* < 0.0001]; VCD-E2 + LEVO: [*F*_(__1_,_17__)_ = 50.17, *p* < 0.0001].

For secondary follicles, there was a Treatment main effect for each group comparison with the ovary-intact Vehicle reference group: (VCD-Vehicle: [*F*_(__1_,_16__)_ = 134.22, *p* < 0.0001]; VCD-E2: [*F*_(__1_,_18__)_ = 175.61, *p* < 0.0001]; VCD-PROG: [*F*_(__1_,_17__)_ = 130.12, *p* < 0.0001]; VCD-LEVO: [*F*_(__1_,_15__)_ = 90.70, *p* < 0.0001]; VCD-E2 + PROG: [*F*_(__1_,_17__)_ = 314.74, *p* < 0.0001]; VCD-E2 + LEVO: [*F*_(__1_,_17__)_ = 141.85, *p* < 0.0001]).

For antral follicles, there was a Treatment main effect for each group comparison with the ovary-intact Vehicle reference group (VCD-Vehicle: [*F*_(__1_,_16__)_ = 40.27, *p* < 0.0001]; VCD-E2: [*F*_(__1_,_18__)_ = 69.44, *p* < 0.0001]; VCD-PROG: [*F*_(__1_,_17__)_ = 46.36, *p* < 0.0001]; VCD-LEVO: [*F*_(__1_,_15__)_ = 47.66, *p* < 0.0001]; VCD-E2 + PROG: [*F*_(__1_,_17__)_ = 62.13, *p* < 0.0001]; VCD-E2 + LEVO: [*F*_(__1_,_17__)_ = 62.13, *p* < 0.0001]).

For corpora lutea, there was a Treatment main effect for each group comparison with the ovary-intact Vehicle reference group: (VCD-Vehicle: [*F*_(__1_,_16__)_ = 263.46, *p* < 0.0001]; VCD-E2: [*F*_(__1_,_18__)_ = 413.27, *p* < 0.0001]; VCD-PROG: [*F*_(__1_,_17__)_ = 184.52, *p* < 0.0001]; VCD-LEVO: [*F*_(__1_,_15__)_ = 102.73, *p* < 0.0001]; VCD-E2 + PROG: [*F*_(__1_,_17__)_ = 278.58, *p* < 0.0001]; VCD-E2 + LEVO: [*F*_(__1_,_17__)_ = 314.74, *p* < 0.0001]).

Interestingly, the ovary-intact vehicle reference group had fewer primary follicles compared to each VCD-treated group: (VCD-Vehicle: [*F*_(__1_,_16__)_ = 41.99, *p* < 0.0001]; VCD-E2: [*F*_(__1_,_18__)_ = 11.85, *p* < 0.01]; VCD-PROG: [*F*_(__1_,_17__)_ = 20.16, *p* < 0.001]; VCD-LEVO: [*F*_(__1_,_15__)_ = 34.13, *p* < 0.0001]; VCD-E2 + PROG: [*F*_(__1_,_17__)_ = 21.74, *p* < 0.001]; VCD-E2 + LEVO: [*F*_(__1_,_17__)_ = 16.46, *p* < 0.001]).

### Body Weights

**FIGURE 14:**
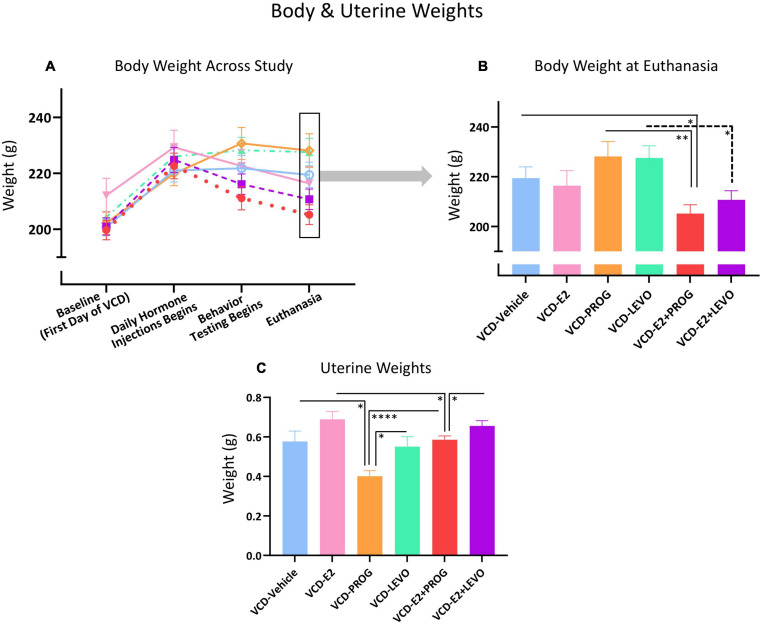
Peripheral markers of overall health and uterine stimulation. **(A)** Body weight changes across the experimental timeline **(B)** At the end of the experiment, the VCD-E2 + PROG group weighed less than the VCD-Vehicle group and the VCD-PROG group, suggesting combination hormone therapy promotes weight maintenance compared to no hormone therapy treatment or progesterone treatment alone. The VCD-E2 + LEVO group also weighed less than its VCD-LEVO alone counterpart, again suggesting combination hormone therapy promotes weight maintenance. **(C)** PROG treatment reduced uterine weight compared to VCD-Vehicle, VCD-LEVO, and VCD-E2 + PROG groups. VCD-E2 + PROG uterine weight was attenuated compared to VCD-E2 treatment along, suggesting progesterone blocked uterine proliferation. The VCD-E2 + LEVO group uteri weighed more than those in the VCD-E2 + PROG group, indicating less progestin-induced attenuation of uterine stimulation compared to natural progesterone when in a combined hormone therapy regimen. Significance: ^∗^ = *p* < 0.05, ^∗∗^ = *p* < 0.01, ^****^ = *p* < 0.0001.

Body Weight measurements across the experiment are illustrated in Figure 14A.

#### How Does Daily E2-Only Treatment Affect Body Weight With Transitional Menopause?

As we have previously observed (Koebele et al., 2020a), there were no body weight differences between the VCD-Vehicle group and the VCD-E2 group at euthanasia, indicating that daily E2 treatment was insufficient to alter body weight compared to a reproductive tract intact, but follicle-deplete, rat not treated with hormone therapy (Figure 14B).

#### How Does Daily Treatment With Progesterone or Levonorgestrel Affect Body Weight With Transitional Menopause, and Does Type of Progestogen Matter?

There were no differences in body weight between the VCD-Vehicle and the VCD-PROG group, or the VCD-LEVO group, at euthanasia. VCD-PROG vs. VCD-LEVO groups did not differ in average body weight either. Overall, this indicates that in reproductive tract intact, follicle-deplete rats, daily progestogen treatment alone did not alter body weight compared to counterparts not treated with hormone therapy. Moreover, body weights from progestogen-only groups did not differ from each other (Figure 14B).

#### How Does Daily Combination Hormone Therapy Affect Body Weight With Transitional Menopause?

The VCD-E2 + PROG group weighed less than the VCD-Vehicle group [*F*_(__1_,_18__)_ = 6.12, *p* < 0.05] as well as less than the VCD-PROG group [*F*_(__1_,_17__)_ = 11.39, *p* < 0.01] at euthanasia. The VCD-E2 + LEVO group weighed less than LEVO-only treated counterparts as well [*F*_(__1_,_17__)_ = 7.84, *p* < 0.05]. However, there were no weight differences indicated between the VCD-Vehicle vs. VCD-E2 + LEVO group at euthanasia. The combination hormone therapy regimens did not have an impact on body weight compared to E2-only treatment, nor did they differ from each other. Overall, these data suggest that a combined hormone therapy regimen, particularly one containing natural progesterone, may lead to weight loss with a follicle-deplete background (Figure 14B).

### Uterine Weights

#### How Does Daily E2-Only Treatment Affect Uterine Weight With Transitional Menopause?

The VCD-Vehicle and VCD-E2 groups did not differ in uterine weight (Figure 14C). Although we have previously reported an increase in uterine weight with E2-only treatment in a VCD model, that experiment administered E2 tonically using Alzet osmotic pumps (Koebele et al., 2020a). It is possible that transitionally menopausal rats given a low dose of E2 via daily injection is insufficient to induce persistent changes in uterine weight compared to transitionally menopausal rats not receiving hormone therapy treatment.

#### How Does Daily Treatment With Progesterone or Levonorgestrel Affect Uterine Weight With Transitional Menopause, and Does Type of Progestogen Matter?

While VCD-Vehicle vs. VCD-LEVO groups did not differ in uterine weights, the VCD-PROG group had decreased uterine weights compared to the VCD-Vehicle group [*F*_(__1_,_17__)_ = 8.14, *p* < 0.05] and compared to the VCD-LEVO group [*F*_(__1_,_16__)_ = 6.92, *p* < 0.05], suggesting that daily natural progesterone treatment attenuates uterine weight in reproductive tract-intact but follicle-deplete rats (Figure 14C).

#### How Does Daily Combination Hormone Therapy Affect Uterine Weight With Transitional Menopause?

Neither combination hormone therapy regimens, E2 plus progesterone nor E2 plus levonorgestrel, had an impact on uterine weight as compared to transitionally menopausal rats without hormone therapy. The combination of E2 plus progesterone decreased uterine weights compared to E2-only treatment [VCD-E2 group vs. VCD-E2 + PROG group: *F*_(__1_,_18__)_ = 5.43, *p* < 0.05], while the combination E2 plus levonorgestrel did not yield this decrease compared to E2-only treatment. Progesterone-only treatment also reduced uterine weights compared to combined E2 plus progesterone treatment [*F*_(__1_,_17__)_ = 31.58, *p* < 0.0001]. Uterine weights did not differ between rats treated with levonorgestrel alone and counterparts treated with a combination of E2 plus levonorgestrel. However, when E2 was administered with levonorgestrel, this combination resulted in higher uterine weights than when E2 was combined with natural progesterone [*F*_(__1_,_18__)_ = 4.627, *p* < 0.05] (Figure 14C).

## Discussion

Using the VCD accelerated follicular depletion model of transitional menopause, this experiment evaluated independent and combined effects of daily E2, progesterone, and levonorgestrel treatment on several aspects of cognition, including spatial memory, anxiety-like, and depressive-like behaviors in middle-aged, ovarian follicle-deplete female rats. Endocrine and ovarian follicular profiles were reported in conjunction with general health measures to provide the first comprehensive report of cognitive outcomes associated with independent and combined menopausal hormone therapy regimens in a transitional menopause model. Until now, preclinical investigations into combined hormone therapy regimens have been conducted in Ovx rats (Gibbs, 2000; Simone et al., 2015; Prakapenka et al., 2018), and evaluations of hormone effects utilizing the VCD model have been limited to estrogen-only (Acosta et al., 2010; Pestana-Oliveira et al., 2018; Long et al., 2019; Koebele et al., 2020a). Divergent cognitive, anxiety-like, and depressive-like profiles were observed dependent upon the type of clinically relevant, daily hormone regimen administered. Overall, under the current experimental parameters, progesterone-only treatment produced detrimental impacts on spatial working memory, while combined E2 plus progestogen treatments resulted in beneficial cognitive effects spanning spatial memory, anxiety-like measures, and depressive-like measures, as well as favorable body and uterine weight profiles in a follicle-deplete, ovary-intact transitional menopause model. Collectively, these findings demonstrate that the presence of follicle-depleted ovarian tissue and the specific formulation of hormone treatment not only yield unique behavioral phenotypes, but are critical considerations when interpreting outcomes in both preclinical and clinical evaluations.

Regarding spatial memory performance, daily E2 treatment in follicle-deplete rats had a neutral effect on working and reference memory compared to counterparts without subsequent hormone treatment. Mode of hormone administration could impact cognitive outcomes in a transitional menopause background. Indeed, we have recently shown that tonic, chronic administration of E2 via a subcutaneous Alzet osmotic pump had beneficial learning effects, and some detrimental memory effects, in follicle-deplete rats of the same age (Koebele et al., 2020a). Thus, although the age of the rats, as well as the VCD treatment, hormone dose, and behavior protocol were constant across studies, varying the drug administration route from a tonic exposure to a daily injection likely altered spatial working memory outcomes.

We also report here that the combined hormone therapy regimen containing E2 plus the synthetic progestin levonorgestrel improved spatial reference memory during task acquisition in the WRAM compared to E2-only treatment. This suggests a unique and broad benefit in the transitionally menopausal model that was not observed in a surgical menopause model, wherein combined E2 plus levonorgestrel treatment attenuated the beneficial effects of E2 alone after Ovx (Prakapenka et al., 2018). Thus, the presence or absence of follicle-deplete ovarian tissue in middle-age plays a role in the cognitive outcomes of E2 plus levonorgestrel combination hormone treatment. Rats that received E2 plus levonorgestrel treatment also had improved reference memory during task acquisition compared to rats treated with E2 plus progesterone concomitantly, suggesting a unique cognitively beneficial role for levonorgestrel when combined with E2 to enhance learning on a complex spatial working memory task. Reference memory benefits observed for the rats treated with daily E2 plus levonorgestrel treatment did not carry over into MM, indicating that the presence of a working memory component in a task alters outcomes on the reference memory measure, an effect we have previously shown in normally aging, ovary-intact rats without hormone treatment (Bernaud et al., 2021). During the latter portion of WRAM testing, transitionally menopausal rats treated with only progesterone showed working memory impairments when working memory was taxed compared to counterparts without hormone treatment, with levonorgestrel treatment, or with E2 plus progesterone treatment. This progesterone-only induced cognitive impairment has been observed in past work from our laboratory and others using the Ovx menopause model (Chesler and Juraska, 2000; Bimonte-Nelson et al., 2006; Harburger et al., 2007; Lowry et al., 2010; Sun et al., 2010; Braden et al., 2015). On the MM, transitionally menopausal rats administered E2-only had significantly better performance on the last day of the task compared to both combination treatment groups, such that in the case of a simple spatial reference memory-only task, the combination of progesterone or levonorgestrel with E2 attenuated performance compared to E2 treatment alone. However, regardless of treatment, all rats spatially localized to the previously platformed area during the probe trial, indicating the effective use of a spatial strategy in the MM. Taken together, the cognitive effects resulting from exogenous hormone treatment may be specific to memory domain, task complexity, and menopause type (Koebele et al., in press). It is also of note that hormone therapy regimens in this study began after follicular depletion was substantial, and cognitive outcomes could have been impacted by the timing of the hormone therapy administration relative to the extent of follicular depletion.

Regarding anxiety-like behavior as measured by the OFT, transitionally menopausal rats treated with a combination of E2 plus levonorgestrel demonstrated less anxiety-like behavior as defined by more time and entries into the open field center compared to E2-only treatment, as well as more entries into the smallest center designation compared to transitionally menopausal rats without hormone therapy, or those given E2-only, or E2 plus progesterone. The E2 plus levonorgestrel group also had increased Total Line Crossings in the OFT, suggesting increased overall locomotor activity with this hormone treatment combination. Increased time in the corners of the open field in the VCD-Vehicle group indicates that the endogenous hormone profile associated with transitional menopause without subsequent hormone therapy increases anxiogenic behavior compared to the profile of transitional menopause with E2-only or progesterone-only administration. This observation corresponds to clinical literature showing increased *de novo* affective disorders during midlife and the transition to menopause, and calls for further evaluations of midlife-aged individuals given these hormone therapies (Bromberger and Kravitz, 2011; Maki et al., 2012; Weber et al., 2014; Soares, 2019; Parry, 2020; Stute et al., 2020). Overall, the combination of E2 and levonorgestrel produced a favorable profile of reduced anxiety-like behaviors compared to other groups. This is particularly noteworthy, as E2-only therapy has been shown to alleviate affective symptoms during the menopause transition, but not in the post-menopausal life stage (Lokuge et al., 2011); perhaps combined hormone regimens could be a novel pathway to alleviate anxiety symptoms in individuals who are reproductive-tract-intact but ovarian follicle-depleted. Regarding depressive-like behavior quantified in the FST, transitionally menopausal rats given combined hormone therapy regimen, irrespective of progestogen type, exhibited longer latencies to immobility and spent less time immobile overall. This suggests that combined hormone therapy regimens, particularly those containing levonorgestrel, produce advantageous outcomes for depressive-like behaviors with a follicle-deplete, ovary-intact background. It is important to acknowledge that traditional FST measures have more recently been discussed within the context of responsiveness or coping after a severe acute stressor, rather than a pure measure of persistent depressive-like behavior (Commons et al., 2017), and that immobility could be an adaptive response rather than a despair-like behavior (Molendijk and de Kloet, 2015). In the future, it will be important to capture the impact of variations in hormone therapy regimens on additional tasks that encompass varied expressions of anxiety-like and depressive-like behavior in rodents.

In terms of physiological measures, all groups treated with E2 had elevated circulating E2 levels compared to groups that were not treated with E2. Circulating progesterone was increased in groups treated with progesterone. Of particular interest, transitionally menopausal rats treated with a combination of E2 and natural progesterone displayed elevated serum progesterone levels compared to counterparts treated with progesterone alone, which may point to a mechanism by which the combined hormone treatment containing E2 plus progesterone increased natural progesterone production to a greater extent than did the exogenous progesterone treatment alone. Circulating androstenedione levels were undetectable in rats treated with E2 alone or in combination with levonorgestrel, suggesting a potential role of exogenous E2 in mediating endogenous androstenedione production, which is synthesized in the interstitial ovarian tissue. Rats treated with progesterone had elevated circulating androstenedione levels compared to counterparts without hormone treatment, with synthetic levonorgestrel, or with combined E2 plus progesterone regimens, indicating that exogenous progesterone alone promotes the synthesis of endogenous androstenedione.

With regard to ovarian follicle counts, we report that the VCD-E2 treated group had significantly fewer primordial and primary follicles compared to the VCD-Vehicle group, corresponding to recent work from our laboratory showing similar effects with tonically administered E2 (Koebele et al., 2020a). This is a novel phenomenon observed within the middle-aged VCD model, wherein exogenous E2-only treatment may further accelerate follicular depletion by a yet-unknown mechanism. One possibility is that exogenous E2-associated rapid follicular depletion may be moderated, in part, by interactions with estrogen receptor-beta (Chakravarthi et al., 2020). Moreover, a recent report in adult ovary-intact mice revealed that administration of the synthetic estrogen ethinyl estradiol downregulated estrogen receptor expression and oxytocin receptor expression in ovarian tissue, with all receptor downregulation persisting even after treatment was discontinued (Garbett et al., 2020), pointing to a role for exogenous estrogen treatment in accelerated follicular depletion in rodents. Interestingly, the VCD-E2 + PROG group had statistically more corpora lutea present compared to the VCD-E2 alone group, such that the group administered E2 only was largely anovulatory, whereas other groups may have had an occasional ovulatory cycle during depletion, as has been observed in individuals during the human menopause transition (O’Connor et al., 2009; Burger, 2011), resulting in quantifiable corpora lutea at the time of evaluation.

The addition of the ovary-intact vehicle reference group confirmed that primordial, secondary, and antral follicles, as well as corpora lutea, were sufficiently depleted in the VCD-treated groups, regardless of subsequent hormone therapy treatment. In contrast to our previously published findings (Koebele et al., 2020a), the ovary-intact vehicle reference group had significantly *lower* primary follicles counts compared to VCD-treated groups. This may be due to a rat strain difference since the F344-NIH strain utilized in our previously published work has since been retired and replaced with the F344-CDF strain. Six single nucleotide polymorphisms (SNPs) that differ between the strains have been detected, although the effect of these SNPs on the F344-CDF phenotypes is not well defined (National Institute on Aging, 2019). Because primary ovarian follicles are not steroidogenic or responsive to gonadotropins, it is unlikely that there would be a major biologically or behaviorally relevant consequence to the increased primary follicle counts observed in the VCD-treated groups herein. It is notable that the extremely low or undetectable numbers of secondary and antral follicles in all VCD-treated groups demonstrate that the ovatoxin successfully halted any remaining primary follicles from transitioning into later stages of growth, and was thus successful at inducing a transitional menopause model.

Combined hormone therapy regimens containing both an estrogen and progestogen appear to reduce or maintain body weight during the menopause transition. Moreover, natural progesterone-only treatment consistently promoted inhibitory effects of uterine proliferation at the dose given. Follicle-deplete rats administered combined E2 plus progesterone therapy showed decreased uterine weights compared to E2-only therapy, again suggesting that natural progesterone administered exogenously attenuated endometrial growth; of note, we also found that progesterone decreased the uterine weight when combined with E2, while the synthetic progestin levonorgestrel did not. A higher dose of levonorgestrel may prevent uterine weight increases with transitional menopause. Of particular clinical relevance, uterine weights from rats treated with either combination hormone regimen did not differ from transitionally menopausal rats without hormone treatment; thus, the tested combined regimens did not yield substantial E2-induced uterine hyperplasia overall.

Collectively, this experiment demonstrates the remarkable variability that hormone therapy options can have on outcomes associated with memory, anxiety, depression, endocrine, body weight, and reproductive tract profiles during the transition to menopause. In accordance with medical societies providing recommendations for care during the menopause transition, our data support the tenet that hormone therapy is not a one-size-fits-all solution (Neves-E-Castro et al., 2015; Stuenkel et al., 2015; Baber et al., 2016; Pinkerton et al., 2017a). Primary indications for treatment and individual health risk factors must be taken into account when prescribing hormone therapy; it is clear that formulation and presence of an intact reproductive tract are key to this equation, despite being historically understudied. The neurobiological, pharmacological, and behavioral effects of E2-alone, progestogen-alone, and combined hormone therapy are complex and, in some cases, task-specific. That levonorgestrel has some androgenic receptor activity, but does not have glucocorticoid or anti-mineralocorticoid activity like natural progesterone or other clinically used progestins (Schindler et al., 2003) may play a role in the behavioral phenotypes observed herein. This is particularly important because progesterone-alone had several negative effects on working memory performance in this evaluation, replicating a well-documented effect in the literature in Ovx rats (Chesler and Juraska, 2000; Bimonte-Nelson et al., 2004, 2006; Harburger et al., 2007; Sun et al., 2010; Braden et al., 2015). Moreover, progesterone-only, but not levonorgestrel-only, treatment increased androstenedione levels in the current experiment. Given that androstenedione has been shown to detrimentally impact spatial memory in Ovx rats, likely via its aromatization to estrone (Camp et al., 2012; Mennenga et al., 2015c), interactive effects of levonorgestrel with androgen receptors in conjunction with lower circulating androstenedione levels than seen with progesterone treatment may be a putative mechanism through which levonorgestrel mitigates or prevents negative cognitive effects. Moreover, levonorgestrel has also been shown to have some unique effects on insulin secretion when combined with the synthetic estrogen, ethinyl estradiol (Sitruk-Ware and Nath, 2011), indicating that independent or combined administration may alter biological and behavioral outcomes. Levonorgestrel remains a popular progestin prescribed in intrauterine devices, combined oral contraceptives, emergency contraception, and menopausal hormone therapy formulations; the results described here are promising findings, as a favorable hormone therapy regimen should not compromise cognitive health for the individual (and optimally would provide benefits) while fulfilling its function to alleviate other non-cognitive, unwanted menopause symptoms. Continued exploration into the biological underpinnings of levonorgestrel’s unique effects on the brain and periphery will provide critical insight for improving health outcomes across multiple stages in the lifespan. Future investigations should consider additional clinically relevant hormone formulations that take into account a more holistic approach to understanding cognitive-behavioral outcomes, including menopause type (Edwards et al., 2019) and individual life history, with the goal to improve healthy life expectancy outcomes.

## Data Availability Statement

The data will be made available by the authors upon reasonable request. Requests to access the datasets should be directed to HB-N, bimonte.nelson@asu.edu.

## Ethics Statement

The animal study was reviewed and approved by Arizona State University Institutional Animal Care and Use Committee.

## Author Contributions

SK contributed to conceptualization, data curation, formal analysis, investigation, methodology, project administration, software, supervision, validation, visualization, writing (original draft), and writing (review and editing). RH contributed to conceptualization, data curation, investigation, methodology, project administration, supervision, validation, visualization, and writing (review and editing). ZP, RM, AP, SP, CC, DK, SM, LM, and CD contributed to investigation, validation, and writing (review and editing). HB-N contributed to conceptualization, data curation, formal analysis, funding acquisition, investigation, methodology, project administration, resources, software, supervision, validation, visualization, writing (original draft), and writing (review and editing). All authors contributed to the article and approved the submitted version.

## Conflict of Interest

The authors declare that the research was conducted in the absence of any commercial or financial relationships that could be construed as a potential conflict of interest.
